# Dependence of Chromatosome Structure on Linker Histone Sequence and Posttranslational Modification

**DOI:** 10.1016/j.bpj.2018.04.034

**Published:** 2018-05-11

**Authors:** Mehmet Ali Öztürk, Vlad Cojocaru, Rebecca C. Wade

**Affiliations:** 1 Molecular and Cellular Modeling Group, Heidelberg Institute for Theoretical Studies (HITS), Heidelberg, Germany; 2 The Hartmut Hoffmann-Berling International Graduate School of Molecular and Cellular Biology, Heidelberg University, Heidelberg, Germany; 3 Computational Structural Biology Laboratory, Department of Cellular and Developmental Biology, Max Planck Institute for Molecular Biomedicine, Münster, Germany; 4 Center for Multiscale Theory and Computation, Westfälische Wilhelms University, Münster, Germany; 5 Center for Molecular Biology (ZMBH), DKFZ-ZMBH Alliance, Heidelberg University, Heidelberg, Germany; 6 Interdisciplinary Center for Scientific Computing (IWR), Heidelberg, Germany

## Abstract

Linker histone (LH) proteins play a key role in higher-order structuring of chromatin for the packing of DNA in eukaryotic cells and in the regulation of genomic function. The common fruit fly (*Drosophila melanogaster*) has a single somatic isoform of the LH (H1). It is thus a useful model organism for investigating the effects of the LH on nucleosome compaction and the structure of the chromatosome, the complex formed by binding of an LH to a nucleosome. The structural and mechanistic details of how LH proteins bind to nucleosomes are debated. Here, we apply Brownian dynamics simulations to compare the nucleosome binding of the globular domain of *D. melanogaster* H1 (gH1) and the corresponding chicken (*Gallus gallus*) LH isoform, gH5, to identify residues in the LH that critically affect the structure of the chromatosome. Moreover, we investigate the effects of posttranslational modifications on the gH1 binding mode. We find that certain single-point mutations and posttranslational modifications of the LH proteins can significantly affect chromatosome structure. These findings indicate that even subtle differences in LH sequence can significantly shift the chromatosome structural ensemble and thus have implications for chromatin structure and transcriptional regulation.

## Introduction

Olins and Olins reported the first electron micrograph of the beads-on-a-string structure of chromatin in 1974 (1). Shortly afterwards, on the basis of biochemical and crystallographic data, Kornberg formulated the nucleosome hypothesis, namely, that in eukaryotes, chromatin consists of repeating units of ∼200 bp DNA wrapped around core histone oligomers connected to form a flexibly jointed chain (2), and this was supported by further electron microscopy evidence (3). Digestion of chromatin by a nonspecific nuclease revealed subnucleosomal particles, chromatosomes, connected by linker DNA (4). Each chromatosome consists of a nucleosome core particle of 147 bp of nucleosomal DNA (N-DNA) coiled around a core histone octamer extended by ∼20 bp of linker DNA (L-DNA) and bound by one linker histone (LH). Thus, the chromatosome can be considered as a fundamental unit of chromatin structure (5). Structurally, LHs are composed of ∼200 amino acid (aa) residues and have three domains: an ∼40 aa unstructured N-terminal tail, a conserved ∼80 aa globular domain (GD), and an ∼100 aa disordered C-terminal tail. Because of the flexibility of the N- and C-terminal tails, only the GD has been crystallized and its structure determined by x-ray crystallography (*Gallus gallus* gH5, Protein Data Bank (PDB): 1HST, 2.6 Å resolution) (6). Despite the recent determination of the crystal structures of LH GD-nucleosome complexes (PDB: 4QLC, 3.5 Å resolution (7); PDB: 5NL0, 5.4 Å resolution (8)), the structural determinants of chromatosome formation are still not well understood. In two studies by Zhou et al. (7, 9), the authors reported that the *G. gallus* LH isoform (gH5) binds on-dyad to a nucleosome with a Widom 601 DNA sequence, whereas the *Drosophila melanogaster* LH globular domain H1 (gH1) binds off-dyad to the same nucleosome. Interestingly, in a follow-up study, by using low-resolution spin-labeling experimental constraints, Zhou et al. (10) suggested that the on-dyad binding mode of the *G. gallus* gH5 to the nucleosome could be switched to an off-dyad binding mode by introducing a pentamutation in the *G. gallus* gH5. These results suggest that it is important to understand the sequence dependence of the structure of the chromatosome, which can have different LH variants and nucleosome sequence combinations.

Various experiments suggest specific effects of LH variants on DNA binding and chromatin condensation. Orrego et al. reported up to 19-fold differences in affinity to chromatin for LH H1 variants (11), and Clausell et al. obtained similar results from atomic force microscopy (12). Brown and colleagues used mutagenesis and fluorescence recovery after photobleaching to map the regions affecting chromatin-binding affinity in H1.1–H1.5 and to identify distinct nucleosome binding surfaces in H1c and H1(0) (13, 14). It was also found that individual LH variants can trigger apoptosis (15) and are differentially expressed during stem cell differentiation, cell cycle progression, and proliferation (16, 17). The specificities and genomic distribution of LH variants was recently reviewed by Kowalski and Palyga (18) and Millán-Ariño et al. (19). These data suggest that LH variants may have distinct functions because of different nucleosome interaction and chromatin compaction mechanisms.

The first posttranslational modification (PTM) of an LH was reported in 1972 (20). Since then, many studies have shown that LHs can have methylation, acetylation, ADPribosylation, ubiquitination, formylation, and PARylation PTMs (21, 22, 23, 24, 25, 26, 27, 28, 29, 30, 31, 32). Izzo and Schneider recently extensively reviewed human and mouse H1 PTMs (33). They reported that H1 phosphorylation can have opposing effects on chromatin condensation. Horn et al. suggested that H1 phosphorylation may regulate ATP-dependent chromatin remodeling enzymes and thus impact chromatin compaction (34). Furthermore, high H1 phosphorylation levels are linked with DNA repair (35), apoptosis (36), cellular aging (37), and cancer events (38). H1 methylation is also associated with heterochromatin organization (39) and cell-cycle-regulated chromatin binding (26). However, although many sites of variant specific PTMs have been characterized, the phenotypic impact of individual LH PTMs is often unknown (40).

**Figure 1:**
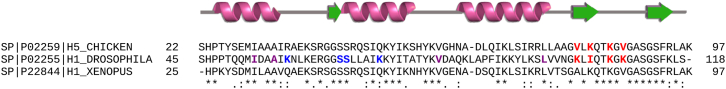
Sequence alignment of the globular domains (GD) of the *G. gallus* H5, *D. melanogaster* H1, and *X. laevis* H1 isoforms. The three LH GD structures have 45% sequence identity. The secondary structure of the GDs is shown above the alignment. Uniprot accession numbers are given at the beginning of each row. Residues that are mutated in *G. gallus* gH5 and *D. melanogaster* gH1 in this work are shown in red. Residues that are posttranslationally modified in *D. melanogaster* gH1 are shown in blue (see Fig. 2). Note that Zhou et al. used a *D. melanogaster* gH1 construct that has core-stabilizing mutations at the residues shown in magenta (9). For consistency, we used the same construct in our simulations for our reference WT *D. melanogaster* gH1. To see this figure in color, go online.

**Figure 2:**
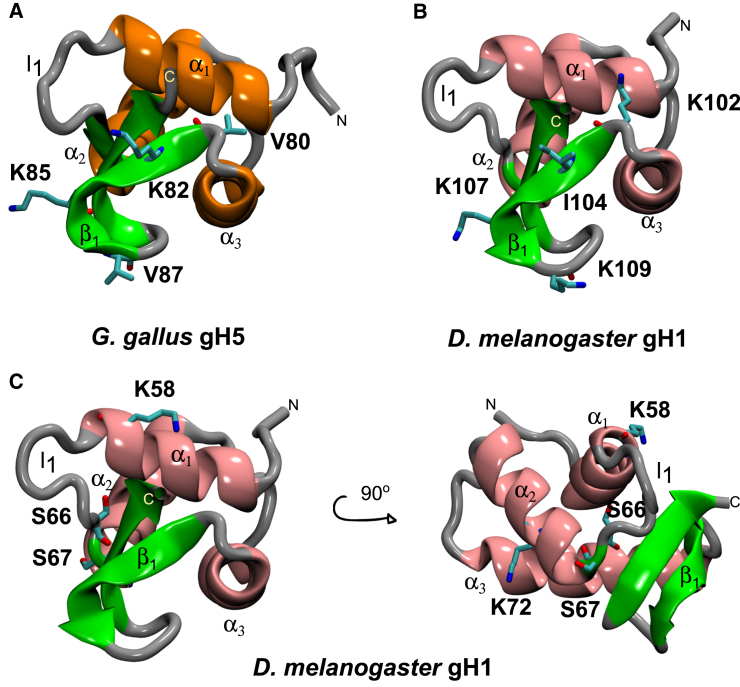
Structures of the LH GDs studied. (*A*) The *G. gallus* gH5 structure, showing the positions of the mutated residues V80, K82, K85, and V87, is given. (*B*) The *D. melanogaster* gH1 structure, showing the positions of the mutated residues K102, I104, K107, and K109, is given. (*C*) The *D. melanogaster* gH1 structure in two orientations, showing the sites of PTMs K72dimethylation, S67phosphorylation, S66phosphorylation, and K58dimethylation, is given. The LH GDs are shown in cartoon representation and colored according to secondary structure: *α*-helices in orange or pink, *β*-sheets in green, and unstructured regions in gray. Mutated side chains are shown in stick representation with coloring by atom type. To see this figure in color, go online.

A range of computational approaches has been used to model and simulate LH-nucleosome complexes. Mesoscale simulations have been applied to explore the influence of LH concentration, conformation, and nucleosome interactions on chromatin structure as well as the dependence of LH-chromatin interactions on salt concentration (41, 42, 43, 44, 45). Most approaches to obtain atomic-detail structures of LH-nucleosome complexes have employed computational docking subject to experimental constraints (46, 47, 48). Most recently, Zhou et al. (9, 10) used HADDOCK (49) and Bednar et al. (8) used Autodock Vina (50) to determine structures of LH GD-nucleosome complexes based on experimental constraints. We have previously shown that Brownian dynamics (BD) rigid-body docking can be used for electrostatically driven macromolecular docking to generate diffusional encounter complexes (51, 52) and could be used without experimental constraints to generate structures of *G. gallus* gH5-nucleosome encounter complexes that were consistent with the available experimental data (53). We then performed atomic-detail molecular dynamics (MD) simulations starting from the BD encounter complexes, which, by taking LH GD and nucleosome flexibility into account, revealed a binding mechanism involving conformational selection and induced fit (54). In the bound complex with an off-dyad position of *G. gallus* gH5, we found that the gH5 *β*_1_-loop V78 makes hydrophobic contacts with the DNA and stabilizes the complex (54). There are exchanges of positive with hydrophobic residues at three positions in the *β*_1_-loop of the LH between *G. gallus* gH5 and *D. melanogaster g*H1 sequences (Figs. 1 and 2), suggesting that mutants with single-point mutations on the *β*_1_-loop could help to understand the determinants of chromatosome structure.

Although atomic-detail and coarse-grained MD simulations have been applied to study the effects of PTMs of core histone tails on protein binding (41, 55), on nucleosome structure (56), and on internucleosome interactions (41), no such studies have yet been reported for variants or PTMs of LHs. In this study, we apply the BD docking approach to investigate the effects of sequence variation and PTMs on the binding configurations of *G. gallus* gH5 and *D. melanogaster* gH1 to the nucleosome. The computational efficiency of the BD approach allows us to consider a number of mutations and PTMs. Moreover, docking is performed for different nucleosome conformations, allowing the relation between LH binding mode and nucleosome opening to be explored. The disordered N- and C-terminal domains of the LH are not included in the models, as it has been shown that they do not affect the location of the GD LH on the nucleosome (7, 8), although the C-terminus affects the affinity (57).

We first validated our BD docking protocol by testing its reproduction of crystallographic structures of LH-nucleosome complexes. We then introduced single-point mutations into both LHs, and by docking the mutants to nucleosome structures, we identified residues that switch chromatosome configurations. Furthermore, we analyzed the effects of *D. melanogaster* gH1 PTMs on LH-nucleosome binding and the distribution of the chromatosome structural ensemble.

## Materials and Methods

We prepared five sets of systems for BD docking simulations (see Table 1). Each system consisted of an LH GD structure and a nucleosome structure to which the LH GD was docked.

**Table 1:** Systems Used in BD Docking Simulations

Nucleosome Structures	DNA Sequence	Core Histones	Number of Nucleosome Conformations for Docking	L-DNA Length (bp)	LH Globular Domain with Conformation in Parentheses	BD Simulations
Crystal structure (PDB: 4QLC, Zhou et al. (7)) and structures from NMA	Widom 601	*D. melanogaster*	3	10	*G. gallus* gH5 (closed)	protocol validation (Fig. 4*A*)

Crystal structure (PDB: 5NL0, Bednar et al. (8)) and structures from NMA	Widom 601L	*X. laevis*	3	26	*X. laevis* gH1 (closed)	protocol validation (Fig. 4*B*)

MD snapshots (Öztürk et al. (54) based on PDB: 1KX5 and 1ZBB	palindromic *H. sapiens* X chromosome *α*-satellite sequence	*X. laevis*	8	10	*G. gallus* gH5 (closed)	gH5 mutants:
						V80K
						K82I
						K85V
						V87K
						(Fig. 5*A*)

MD snapshots (Öztürk et al. (54) based on PDB: 1KX5 and 1ZBB	palindromic *H. sapiens* X chromosome *α*-satellite sequence	*X. laevis*	8	10	*D. melanogaster* gH1 (same as Zhou et al. (9))	gH1 mutants:
						K102V
						I104K
						K107
						K109V
						(Fig. 5*B*)

MD snapshots (Öztürk et al. (54) based on PDB: 1KX5 and 1ZBB	palindromic *H. sapiens* X chromosome *α*-satellite sequence	*X. laevis*	8	10	*D. melanogaster* gH1 (same as Zhou et al. (9))	gH1 PTMs:
						K58 dimethylation
						S66 phosphorylation
						S67 phosphorylation
						K72 dimethylation (Fig. 5*C*)

The five different simulation systems and the details of their structural components are given. See Fig. S1 for a comparison of the three different DNA sequences in the nucleosomes studied.

### LH GD-nucleosome structures

Recently, two crystal structures were reported for LH GD-nucleosome complexes (PDB: 4QLC (7) and 5NL0 (8)). To confirm the validity of our computational protocol, we used these structures as control test systems. From each structure, we created two PDB files, one for the nucleosome and one for the LH GD. Conformational variability of the nucleosome was considered as done previously (53) by generating a set of structures by performing an elastic network normal mode analysis (NMA) using the NOMAD-Ref server (58). For the nucleosome structure from PDB: 5NL0, the following parameters were used so as to generate structures with slightly more open L-DNA arms than the crystal structure: number of modes to calculate, 106; distance weight parameter for elastic constant, 5 Å; elastic network model cutoff for mode calculation, 10 Å; average root mean-square deviation (RMSD) in output trajectories from the initial structure, 3 Å; and calculation method, all-atom and automatic. For the nucleosome from PDB: 4QLC, we used the same nucleosome structures obtained with the default NOMAD-Ref server parameters as used in our previous study (54), which were number of modes to calculate, 16; distance weight parameter for elastic constant, 5 Å; elastic network model cutoff for mode calculation, 10 Å; average RMSD in output trajectories from the initial structure, 1 Å; and calculation method, all-atom and automatic. The output structures of the nucleosomes were named mode 7_0_ (crystal structure), mode 7_1_, and mode 7_2_ and correspond to snapshots along the lowest frequency mode (mode 7, modes 1–6 correspond to rigid body translation and rotation) with increasingly more open L-DNA arms.

As homology-modeled LH structures were used to fit the LH densities in the recent crystal structures, the LH structures extracted from these PDB files were refined using the GalaxyRefine web server tool (59) to increase the structural quality of the side chains of the LHs by using the “mild relaxation only” option. The GalaxyRefine tool rebuilds side chains and performs side-chain repacking and structure relaxation with an MD-simulation-based protocol. The tool ranked best for improving the local structure quality in the CASP10 assessment (59). In all refinements, the all-atom RMSD of the input and output LH structures of the GalaxyRefine tool were below 2 Å.

### Apo-nucleosome structures

The eight snapshots from the MD simulation of an apo-nucleosome performed by Öztürk et al. (54) and previously used for BD rigid-body docking simulations were used. Öztürk et al. showed that the BD docking to these snapshots resulted in similar LH binding configurations to those obtained for nucleosome structures generated by NMA by Pachov et al. (53). Here, we used our previously generated snapshots of nucleosome structures from MD simulations rather than NMA, as MD provides more realistic structures than NMA, albeit at greater computational cost. These nucleosome structures were derived from the crystal structures with PDB: 1KX5 (1.9 Å resolution) (60) for the nucleosome core particle and with PDB: 1ZBB (9 Å resolution) (61) for the 10 bp extensions of each L-DNA. The following procedure was previously used (54): the N-DNA was extended with two L-DNA arms and core histone tails were removed. Nucleosome dynamics were simulated for 100 ns by standard MD simulation. After clustering of structures from the trajectory, eight different snapshots were selected to cover the conformational space of the nucleosome, in which the L-DNA2 arm was in a highly populated conformation and the conformation of the L-DNA1 arm varied (for details of the nucleosome structures, see Figs. S2 and S3; Table 1; (54)). The eight nucleosome structures have different L-DNA1 arm opening and closing angles: snapshots 6, 7, and 8 have a more open L-DNA1 arm, and snapshots 1, 2, 3, and 4 have a more closed L-DNA1 arm compared to snapshot 5 (see Figs. S1–S3; Table 1).

### LH GD structure

The refined *G. gallus* gH5 crystal structure (PDB: 1HST, chain B (6)) was used for docking to the apo-nucleosome structures. Exchanges of positive and hydrophobic residues at four positions in the *β*_1_-loop of the *G. gallus* gH5 and *D. melanogaster g*H1 sequences were selected (Figs. 1 and 2 *A*). The V80K, K82I, K85V, and V87K mutations were introduced individually into *G. gallus* gH5 to construct single-point mutants. The structure of *D. melanogaster* gH1 as reported by Zhou et al. (2013) (9) was kindly provided by Yawen Bai, and the K102V, I104K, K107V, and K109V mutations were introduced into *D. melanogaster* gH1. All mutations were introduced using the PyMOL molecular modeling software (62) (Figs. 1 and 2, *A* and *B*), and then each structure was refined using the GalaxyRefine structure refinement web server tool (59) as described above.

Additionally, the K72dimethylation, S67phosphorylation, S66phosphorylation, and K58dimethylation PTMs (Fig. 2 *C*) (26) were introduced into the *D. melanogaster* gH1 by applying the PyTMs plugin in PyMOL (62, 63) to the refined wild-type (WT) structure. As the GalaxyRefine web server only accepts standard amino acids, partial atomic charges and radii of the posttranslationally modified residues were obtained from previously published studies (64, 65) and added manually to the PQR files generated for these structures without further refinement.

### BD preparation and simulation parameters

For BD simulations, polar hydrogen atoms were added to the structures by using the PDB2PQR 2.1.1 web-server (66), and partial atomic charges and atomic radii were assigned by using the AMBER99 force field (67). For all structures, the molecular electrostatic potentials were calculated by using APBS 1.4 (68) to solve the nonlinear Poisson-Boltzmann equation with a 1 Å grid spacing. Input parameters were a temperature of 298.15 K, solvent- and solute-relative dielectric constants of 78.54 and 2, respectively, and an ionic strength of 100 mM. The van der Waals surface was used to define the dielectric boundary. Effective charges were assigned to charged residues on the protein and to P atoms on the DNA using the Effective Charges for Macromolecules (ECM) program (69). BD simulations were performed by using the SDA 7 software (70) with electrostatic interaction forces and neglecting short-range interactions. The solutes diffused as rigid bodies and overlap between the GD LH and the nucleosome was prevented by applying a 0.5 Å excluded volume criterion. The BD trajectories were started with the geometric center of LH GD positioned randomly on a sphere centered on the nucleosome at a center-to-center distance of ∼185 Å and stopped at a center-to-center distance of ∼204 Å. A time step of 1 ps was used. For each system, we generated 20,000 BD trajectories, and our test runs for 10,000 BD trajectories and for different initial random-number seeds resulted in similar cluster configurations and population percentages. The following two geometric conditions were used to define formation of the diffusional encounter complexes: 1) the geometric center-to-center distance of LH and the nucleosome <98 Å, and 2) the nucleosome dyad point and LH center separation <40 Å. The coordinates and interaction energies of a complex at a given time step were recorded if the RMSD to the previously recorded complexes was >1 Å and the interaction energy was within the 5000 most favorable energy complexes recorded. A complex with RMSD <1 Å to a previously recorded complex but lower energy was recorded as a substitute of that complex; higher energy complexes were added to the count of occurrence of the closest recorded complex with lower energy. Finally, we clustered the top 5000 lowest energy structures into 10 groups, which were ranked according to cluster size, taking the number of counts for each complex recorded into account (for details see (54)).

### Analysis of docked encounter complexes

**Figure 3:**
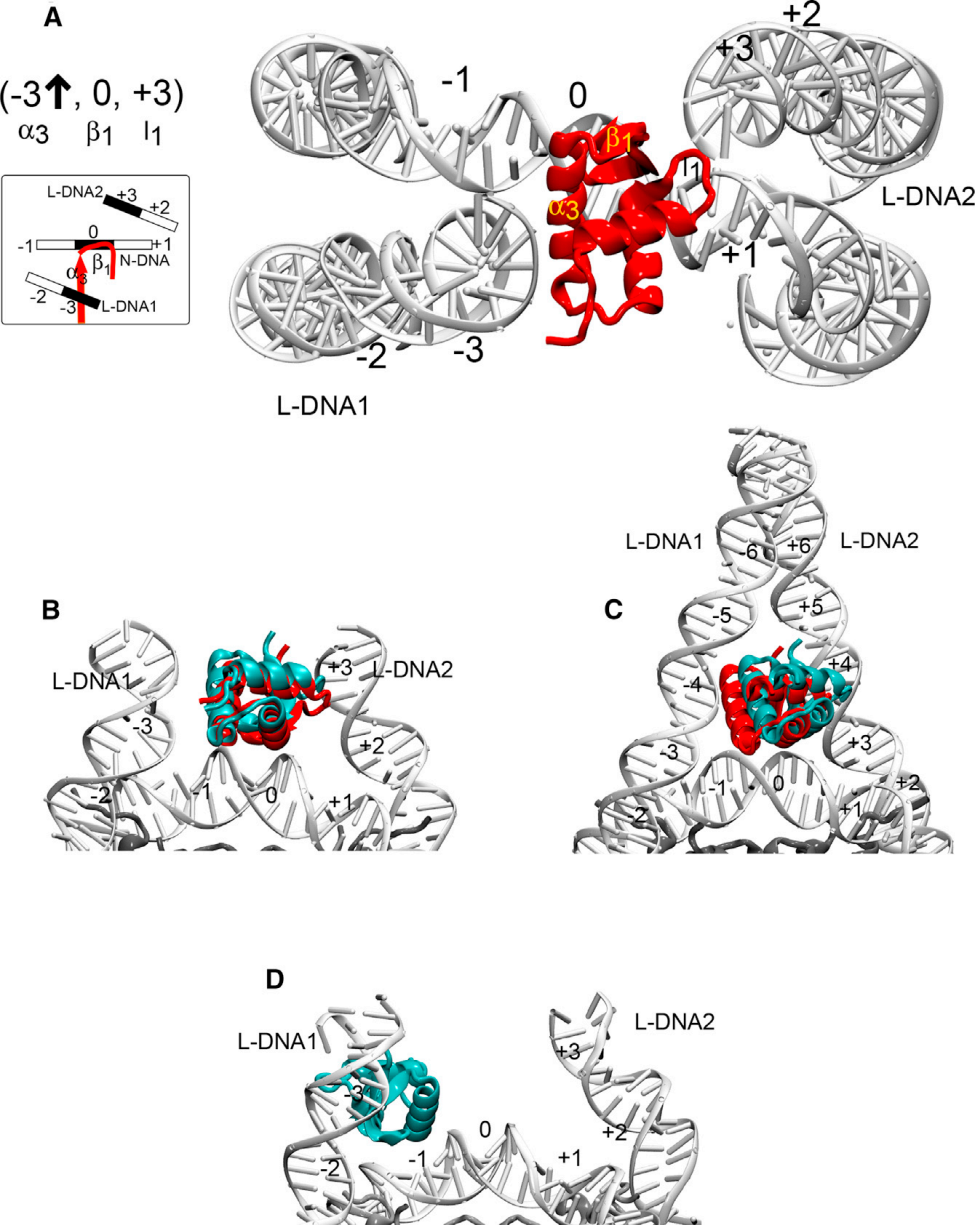
Representative LH-nucleosome encounter complexes from BD docking simulations. LHs are shown in cartoon representation and shown in red for reference crystal structures and cyan for docking results. (*A*) The crystal structure of the complex formed by *G. gallus* gH5 bound to a 147 bp Widom 601 DNA sequence nucleosome (PDB: 4QLC) (7) is shown. The classification of the configuration as (−3↑, 0, +3) (on-dyad) is illustrated. (*B*) A representative structure from the largest diffusional encounter-complex cluster (cluster 1) from the docking of *G. gallus* gH5 (residues 24–98) to the mode 7_1_ structure of the nucleosome derived by NMA from the crystal structure PDB: 4QLC (7) shown in (*A*). Compared to the position in the crystal structure (*red*), the gH5 has a C*α* RMSD of 3.6 Å and the same docked on-dyad configuration (−3↑, 0, +3). (*C*) A representative structure from encounter-complex cluster 2 for *X. laevis* gH1 docked to the nucleosome structure from PDB: 5NL0 (8) is shown. Compared to the position in the crystal structure (*red*), the gH1 has a C*α* RMSD of 5.5 Å and the same docked on-dyad configuration (−3↑, 0, +3). (*D*) A representative structure from the encounter-complex cluster with the greatest population (cluster 1) from docking WT *G. gallus* gH5 to snapshot 5 from MD simulation of the nucleosome (see Table 1; Table S6), which represents the average structure in the simulation. The docked configuration is (−1 ↘, −1 −2, −2) and off-dyad. To see this figure in color, go online.

The configuration of the LH on the nucleosome was classified for the representative structures of the first two largest clusters of encounter complexes with the highest populations obtained in each docking simulation by applying the following procedure. The nucleosome dyad axis was aligned perpendicular to the viewing plane and the DNA grooves were labeled. The minor groove on the dyad was labeled as 0, and the neighboring major grooves of N-DNA toward L-DNA1 and L-DNA2 were labeled as −1 and +1, respectively. The adjacent major grooves on the L-DNA1 and L-DNA2 were labeled as −2 and +2, respectively, and so on to the ends of the L-DNA arms. The DNA groove contacts of the structural elements of the LH (*α*_3_, *β*_1_, and l_1_) were computed for the representative structure of each docking cluster and represented by a vector (see Fig. 3 *A*). The orientation of the *α*_3_-helix of LH was determined, and an arrow was added to the vector to represent the direction of the vector from the N- to the C-terminus of the *α*_3_-helix. An X sign was used when the *α*_3_-helix vector was perpendicular to the viewing plane. See Fig. 3 *A* for an example of the analysis for the crystal structure PDB: 4QLC in the configuration (−3↑, 0, +3). The PyMOL software (62) was used to quantify hydrogen bonding (with a distance criterion of 3.2–3.6 Å) between the LH and the nucleosome structure.

## Results and Discussion

### BD simulations recapitulate experimentally determined LH-nucleosome complex configurations

First, we tested the ability of our protocol of structural refinement of the LH followed by BD rigid-body docking to reproduce the experimentally determined LH-nucleosome structures. In this comparison, it should be borne in mind that the docking protocols generate diffusional encounter complexes that are expected to be close to but not identical to the bound structures studied experimentally. In particular, the rigid-body docked complexes are expected to be looser and will lack optimization of short-range hydrogen bonds and hydrophobic contacts. Therefore, we compared the structures using a classification of the binding configurations based on LH-nucleosome contacts rather than commonly used measures based on RMSD.

**Figure 4:**
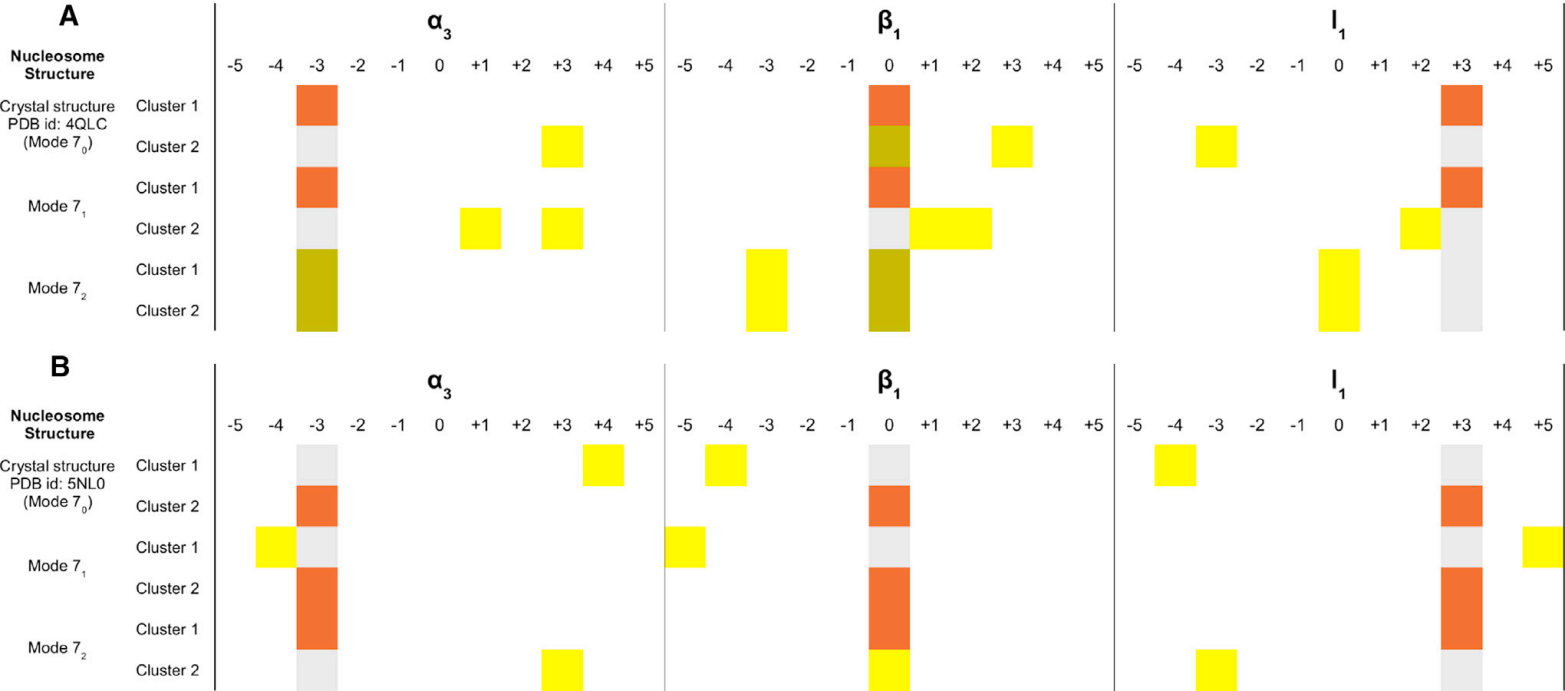
Comparison of BD-docked LH GD-nucleosome encounter complexes with crystal structures of the complexes. (*A*) The docking of WT *G. gallus* gH5 to the nucleosome (PDB: 4QLC, Zhou et al. (7)) is shown. (*B*) The docking of WT *X. laevis* gH1 to the nucleosome (PDB: 5NL0, Bednar et al. (8)) is shown. For each system, the orientations of the representative structures of the largest two clusters of docked encounter complexes are given for docking of the LH GD to the nucleosome crystal structure (mode 7_0_) and two structures generated by normal mode analysis with slightly opened L-DNA arms (modes 7_1_ and 7_2_) (see Materials and Methods and Table S2 for details). The DNA grooves on the nucleosome in contact with each structural element of the LH (*α*_3_, *β*_1_, and l_1_) are given in the respective columns (see Fig. 3). Color code: the DNA groove contacts of the *α*_3_, *β*_1_, and l_1_ elements are given in gray for the crystal structures, in orange when BD results match with the DNA groove contacts of the crystal structures for all three structural elements, in green when the BD results partially (only for one or two structural elements) match with the crystal structures, and in yellow when the BD results have different DNA contacts from the crystal structures. For encounter-complex cluster populations, see Table S3. To see this figure in color, go online.

Zhou et al. (2015) published the crystal structure of *G. gallus* gH5 bound to a nucleosome with a Widom 601 sequence (PDB: 4QLC) (7). This crystal structure shows an on-dyad binding mode of the gH5. In the current docking simulations and in our previous BD docking study (54), WT *G. gallus* gH5 binds to the nucleosome from the 4QLC structure in an on-dyad configuration. The orientation of gH5 corresponds to that in the crystal structure in the largest encounter-complex cluster (cluster 1) obtained by docking gH5 to the nucleosome of the chromatosome crystal structure PDB: 4QLC (mode 7_0_) and to the slightly more open mode 7_1_ structure (see Figs. 3 *B* and 4 *A*; Table S3). It should be noted that for the same system in our previous docking simulations (54), we did not apply an LH refinement protocol, and some opening of the nucleosome, as represented by the mode 7_1_ and mode 7_2_ snapshots, was necessary to allow access of the LH to the nucleosome dyad axis and to reproduce the crystallographic binding mode. This opening of the nucleosome was not necessary for the refined LH structure to bind in the crystallographic binding mode, although binding in this orientation was facilitated by the slight opening in the mode 7_1_ structure.

Bednar et al. published the crystal structure of *Xenopus laevis* gH1 bound to a palindromic Widom 601L nucleosome (PDB: 5NL0) (8). This crystal structure also shows an on-dyad mode for gH1. Application of our LH refinement and docking protocol reproduced the configuration observed in the crystal structure (−3 ↑, 0, +3) in the first- or second-largest encounter-complex cluster when using any of the three nucleosome conformations (modes 7_0_–7_2_) (Figs. 3 *C* and 4 *B*). Interestingly, the number of encounter complexes observed in each docking simulation was somewhat lower than for the gH5 simulations (1–1.5 million compared to 1.4–2.0 million; see Table S3), indicating that the LH binding site was less accessible, possibly because of the longer L-DNA arms (26 vs. 10 bp). Consistently, in the docking simulations of *X. laevis* gH1 to the crystal structure (mode 7_0_) and mode 7_1_ of the nucleosome, the *α*_3_-helix of the LH binds to the L-DNA grooves +4 and −4, respectively (see Table S3). When the L-DNA arms open further in the mode 7_2_ nucleosome structure, the LH can approach closer to the LH core, and the LH *α*_3_-helix binds predominantly to L-DNA groove −3, as observed in the crystal structure. This indicates that further conformational relaxation of the LH and nucleosome should stabilize these on-dyad binding modes.

Summarizing, the diffusional encounter-complex structures generated by BD docking simulations are largely consistent with the crystallographic results of Zhou et al. (7) and Bednar et al. (7, 8) for two different LH-nucleosome systems (Fig. 4). We also previously obtained both on- and off-dyad LH binding modes consistent with the available experimental data by BD docking simulations using nucleosome structures generated by normal mode analysis and by MD simulation (53, 54). We therefore applied the BD docking approach to investigate the effects of mutations and posttranslational modifications on LH-nucleosome binding configurations.

### Single-point mutations in the LH globular domain can significantly affect chromatosome structure

**Figure 5:**
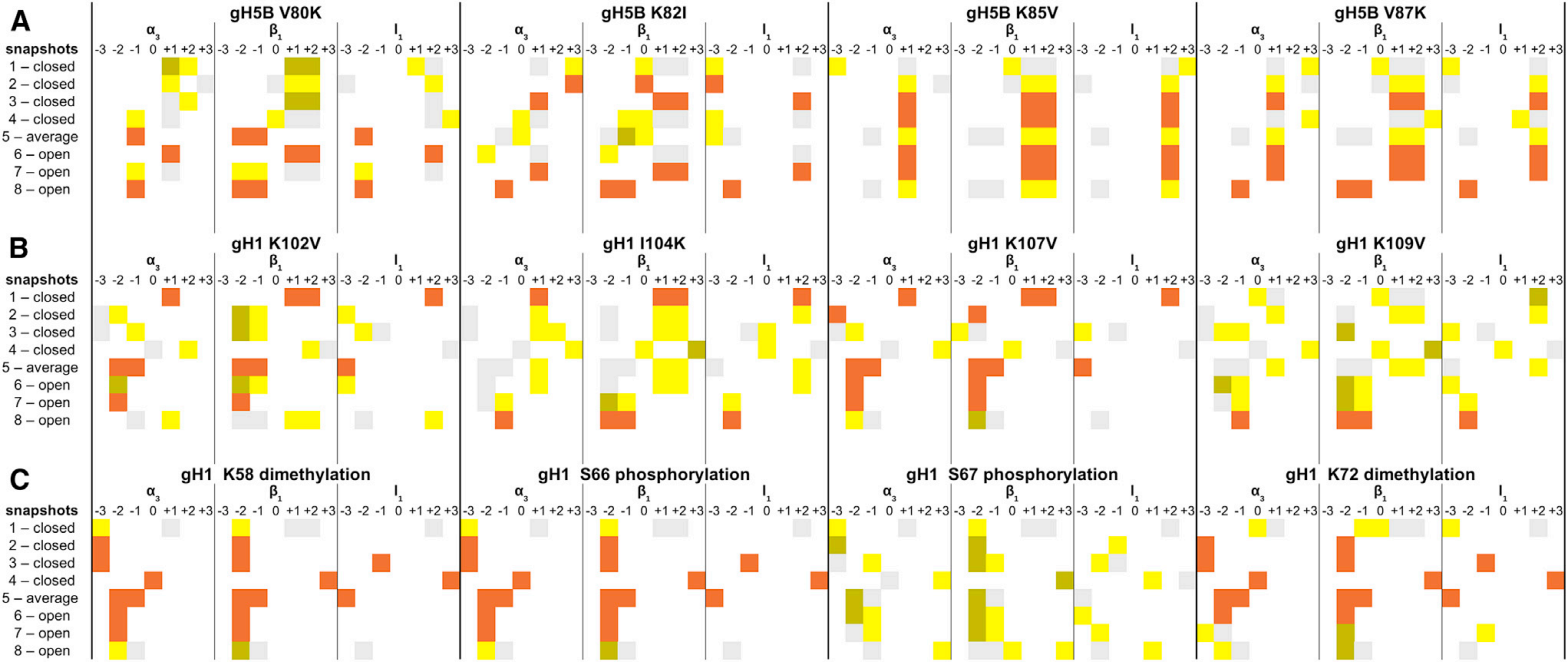
Comparison of the BD-docked configurations of *G. gallus* gH5 and *D. melanogaster* gH1 variants to those for WT LH GDs upon docking to eight representative nucleosome structures. (*A*) WT and mutant *G. gallus* gH5, (*B*) WT and mutant *D. melanogaster* gH1, (*C*) and WT and posttranslationally modified *D. melanogaster* gH1 are all shown. The figures show the orientations of the representative structures of the largest cluster of encounter complexes from docking each LH GD to eight representative structures of the nucleosome from an MD simulation started from PDB: 1KX5 (57) (Table 1). The coloring scheme is the same as in Fig. 4. For cluster populations, see Tables S6–S8. To see this figure in color, go online.

BD docking results for *G. gallus* gH5 and *D. melanogaster* gH1 binding to the eight representative nucleosome structures generated by MD simulation (Figs. S2 and S3) are given in Fig. 5. The nucleosome structures open and close the L-DNA arms to different extents, which were sampled by MD simulation. With respect to the apo-nucleosome structure (snapshot 5), snapshots 1, 2, 3, and 4 are more closed structures, and snapshots 6, 7, and 8 are more open structures (see Figs. S2 and S3 (54)). The binding mode of the largest encounter-complex cluster obtained for the LH variants is compared with that for the WT LH GD for each of the nucleosome structures (see Tables S6 and S7 for the results for the two largest encounter-complex clusters and their populations). Mutant LH-nucleosome configurations that differ significantly from the configurations of the WT LH GD are highlighted in yellow, whereas those that are conserved are highlighted in orange in Fig. 5; gray indicates the configuration obtained from docking the WT LH GD, and green indicates a partial configuration similarity (only for one or two structural elements) with the WT LH.

As found before (53, 54), all docked configurations of WT gH5 (with a closed loop) to eight nucleosome structures are off-dyad and in the (−1↘, −1 −2, −2) configuration (Fig. 3 *D*) for the nucleosome structure close to the crystal structure, snapshot 5, and the most open structure, snapshot 8 (Figs. 3 *D* and 5 *A*). The off-dyad LH binding mode is similar to that identified from NMR (9, 10) and cryo-electron microscopy (71) experiments. For the other snapshots, the alternative off-dyad docking position is similar to L-DNA1 binding configuration (−1↘, −1 −2, −2), but binding to L-DNA2 (+1↖, +1 +2, +2) dominates, as observed previously (54).

For the gH5 mutants, both off-dyad and on-dyad configurations are observed when considering all eight nucleosome snapshots (Fig. 5 *A*). Interestingly, the on-dyad binding of WT and mutant *G. gallus* gH5 is not observed in the more open nucleosome conformations, suggesting that L-DNA opening is important for the distinction between off- and on-dyad binding modes of the LH. Additionally, the *G. gallus* gH5 K82I mutation resulted in four on-dyad binding modes out of a total of eight docking simulations (see *β*_1_-loop contacts of K82I with DNA groove 0 in Fig. 5 *A*). Considering that the gH5 K82 residue is located at the beginning of the *β*_1_-loop of the LH (Fig. 2 *A*), the unit charge reduction resulting from the isoleucine substitution could reduce contact with the L-DNA arms, making the on-dyad configuration more preferable for this mutant. Moreover, hardly any *β*_1_-loop binding to L-DNA1 is observed for WT and mutant gH5 docking to the more closed nucleosome snapshots. For the most open conformers (snapshots 7 and 8), mainly the off-dyad mode is observed because of the opening of the L-DNA1 arm. Remarkably, mutation of gH5 K85 (which is conserved as lysine at the corresponding position in *D. melanogaster* gH1 and *X. laevis* H1) to valine revealed an off-dyad L-DNA2 binding mode (+1↖, +1 +2, +2) in seven out of eight docking simulations, indicating that, in addition to L-DNA opening angles, LH sequence is also a determinant of the binding configuration.

WT *D. melanogaster* gH1 overall adopts a greater diversity of bound configurations than WT *G. gallus* gH5, but all are off-dyad except for the on-dyad docking mode observed for the most closed nucleosome structure, snapshot 4, and for the docking of the K109V mutant to a closed nucleosome structure, snapshot 1 (Fig. 5 *B*). Interestingly, all gH1 mutants, except K102V, bind on-dyad to snapshot 4. Furthermore, compared to the WT and mutant gH5 simulations, more of the docking poses for WT and mutant gH1 display off-dyad binding to the L-DNA1 arm (Fig. 5, *A* and *B*). These results suggest that *G. gallus* gH5 and *D. melanogaster* gH1 have distinct nucleosome binding preferences. For most snapshots, the number of encounter complexes recorded is lower for gH1 than gH5 (see Tables S6 and S7), indicating lower accessibility to the nucleosome, which also correlates with the higher accessible surface area of gH1 compared to gH5 (3998 vs. 3810 Å^2^). For the most open structure, snapshot 8, both gH1 and gH5 bind predominantly in the same off-dyad (−1 ↘, −1 −2, −2) configuration (Fig. 5, *A* and *B*).

The effect of LH mutations on the LH GD-nucleosome complex configuration varies among the different snapshots of the nucleosome. For the *G. gallus* gH5 mutants, docking to nucleosome snapshots 1, 2, 4, and 5 resulted in a major configuration shift compared to *G. gallus* gH5 WT for all the mutants (more yellow and less orange in the rows in Fig. 5 *A*). For the *D. melanogaster* gH1 mutants, the LH configuration was most affected (with shifts for all four mutants, more yellow and less orange in the rows in Fig. 5 *B*) compared to WT *D. melanogaster* gH1 for nucleosome snapshot 4, the snapshot with the most closed conformation of the nucleosome. On the other hand, for some snapshots, there were very few shifts in LH-nucleosome configuration upon mutation. For *G. gallus* gH5, only one mutant showed a shift for nucleosome snapshots 6, 7, and 8 (Fig. 5 *A*), whereas for *D. melanogaster* gH1, only one mutant showed a shift in nucleosome snapshot 1 (Fig. 5 *B*). The results show that point mutations may result in a range of changes to LH-nucleosome binding configuration that are dependent on L-DNA opening. The results for the gH5 mutants indicate that chromatosome formation for the more open nucleosome structures may be less sensitive to gH5 sequence, which would have implications for LH binding mechanisms in chromatin, the formation of chromatin structure, and the phenotypic effects of mutations on LHs.

The applied point mutations involved either the introduction or the removal of a +1 charge from the total +11e charge of the two LHs by the exchange of a lysine residue with a hydrophobic residue. Each single-point mutation had a significant effect on LH docking to at least one of the eight different nucleosome structures. This observation is consistent with the idea that LH-nucleosome recognition is strongly affected by electrostatic interaction forces. For *G. gallus* gH5, the total number of configuration changes (number of rows with contacts shown in yellow and green indicating, respectively, no or partial overlap with the WT LH configuration) in the first encounter-complex clusters for docking to the eight different nucleosomes are 5 (V80K) and 4 (K85V, K82I, and V87K), whereas for *D. melanogaster* gH1, they are 7 (K109V), 6 (I104K), 5 (K102V), and 5 (K107V). Previously, by using BD and MD simulations, we showed that *G. gallus* gH5 V87 makes hydrophobic contacts with nucleosome thymine methyl groups in the off-dyad binding mode that are enhanced by induced fit and the adoption of a loop-out conformation of the gH5 (54). Although the rigid-body docking results presented here indicate that the V80, K82, K85, and V87 residues of the *G. gallus* gH5 and the corresponding K102, I104, K107, and K109 residues of *D. melanogaster* gH1 are important for nucleosome recognition, we anticipate that the mutations will also affect stabilization of the chromatosome complex by induced fit.

**Figure 6:**
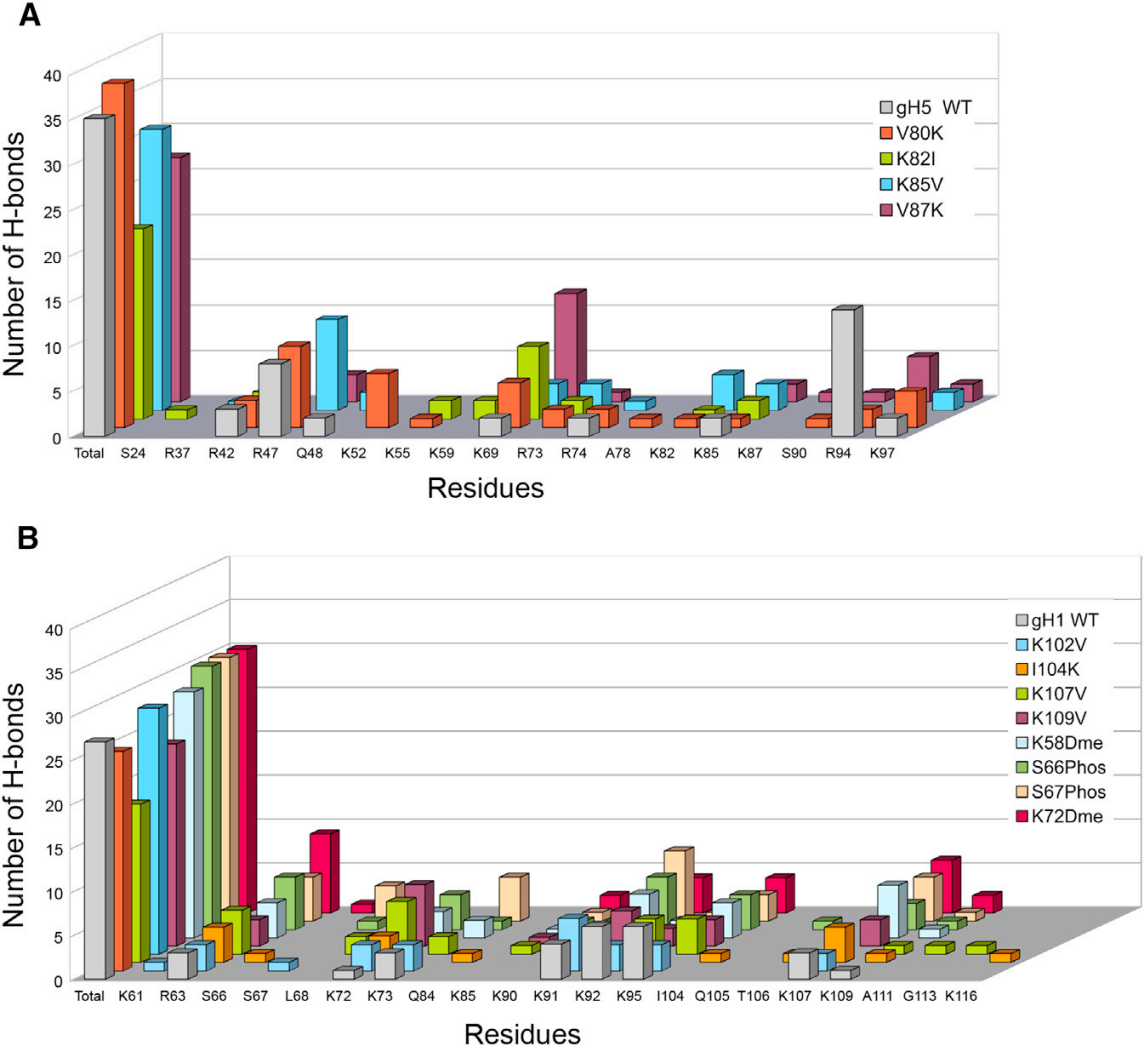
The number of H-bonds formed between nucleosomal DNA and LH GDs. The number of H-bonds formed between nucleosomal DNA and (*A*) WT or mutant *G. gallus* gH5 or (*B*) WT, mutant, or posttranslationally modified *D. melanogaster* gH1 are given, summed up over the eight different LH GD-nucleosome docking simulations (each with a different nucleosome conformation) for each LH variant. Some residues make more than one H-bond in the docked position. To see this figure in color, go online.

Analysis of hydrogen bonds between the LH and the phosphate backbone of the DNA indicates that WT and mutant *D. melanogaster* gH1 generally make fewer hydrogen bonds in the encounter complexes compared to WT and mutant *G. gallus* gH5. Summing up the eight different docking simulations and the two largest encounter-complex clusters (Tables S6–S8), WT *D. melanogaster* gH1 makes 27 hydrogen bonds, whereas *G. gallus* gH5 makes 35 hydrogen bonds (Fig. 6; Tables S4 and S5). Interestingly, in WT *D. melanogaster* gH1, the residues making the most hydrogen bonds are K92 and K95 on the *α*_3_-helix, which can bind to alternative DNA grooves on the nucleosome (Fig. 6; Table S5). On the other hand, in *G. gallus* gH5, most of the hydrogen bonds formed in docking simulations are made by R47 and R94 on the *α*_2_-helix and *β*-sheet, respectively (Fig. 6; Table S4). These hydrogen-bonding differences indicate that different LH isoforms may have different nucleosome recognition mechanisms.

The introduction of single-point mutations in the LHs also resulted in significant changes in hydrogen bonding with the nucleosome. Summing up the eight nucleosome structures of the two largest encounter-complex clusters (Tables S6 and S7), the residues that make more than six hydrogen bonds with the nucleosome in *G. gallus* gH5 mutants are R47 (9 hydrogen bonds (H-bonds) made by V80K mutant and 10 H-bonds made by K85V mutant), K52 (6 H-bonds made by V80K mutant), and K69 (8 H-bonds made by K82I mutant and 12 H-bonds made by V87K mutant) (Fig. 6; Table S4). For *D. melanogaster* gH1 docking, the corresponding residues are K72 (6 H-bonds made by K107V mutant and 7 H-bonds made by K109V mutant) and K91 (6 H-bonds made by K102V mutant) (Fig. 6; Table S5). These results indicate that the hydrogen-bonding network of LH-nucleosome interaction is sensitive to point mutations. Remarkably, the I104K mutant of *D. melanogaster* gH1 makes far fewer H-bonds (18 in eight simulations) compared to the other mutants and PTMs (Fig. 6; Table S5). Interestingly, a significant shift in configuration for *D. melanogaster* gH1 (six of eight encounter complexes shifted compared to WT) is also observed for this mutant, suggests that H-bonding is important for the LH-nucleosome configuration.

In certain LH mutant and nucleosome combinations, single-point mutations on the LH are able to switch the LH binding mode from *D. melanogaster* gH1 to WT *G. gallus* gH5 or vice versa. For example, in docking the I104K mutant of *D. melanogaster* gH1 to nucleosome snapshots 1 and 6, the representative structures from the largest encounter complexes are similar to the configurations for WT *G. gallus* gH5 docking to the same nucleosome conformations (+1 ↖, +1 +2, +2) (Fig. 5, *A* and *B*). Similarly, docking the K109V mutant of the *D. melanogaster* gH1 to nucleosome snapshot 6 yielded similar configurations to WT *G. gallus* gH5 for docking to the same nucleosome conformations (+1 ↖, +1 +2, +2) (second encounter complex; Tables S6 and S7). In addition, docking of the K82I mutant of the *G. gallus* gH5 to nucleosome snapshot 6 (cluster 1: (−2 ←, −2, no)) resulted in similar configurations to WT *D. melanogaster* gH1 (Fig. 5, *A* and *B*; Tables S6 and S7). These results indicate that single-point mutations may switch the chromatosome configurations of different LH isoforms.

Even though the sequences of *D. melanogaster* gH1 and *G. gallus* gH5 share 49% sequence identity (Fig. 1), both WT LH GDs docked in the same off-dyad mode to the most open nucleosome conformation, snapshot 8, as (−1 ↘, −1 −2, −2) (Fig. 5, *A* and *B*; Tables S6 and S7). This shows that, apart from the amino-acid sequence of the LH, L-DNA opening of the nucleosome affects the chromatosome configuration. It also suggests that open nucleosome conformations may be able to bind LH proteins off-dyad nonspecifically, and that subsequently more specific on- and off-dyad configurations are formed upon LH-induced nucleosome closing.

### PTMs of *D. melanogaster* gH1 can modulate LH-nucleosome binding

In addition to single-point mutations, our docking results revealed that PTMs can also switch the configuration of *D. melanogaster* gH1-nucleosome binding. Four known PTMs, two lysine dimethylations and two serine phosphorylations, were investigated. Dimethylation interferes with salt-link formation, and phosphorylation introduces negative charge. The number of significant shifts in chromatosome configurations (Fig. 5 *C*) upon introducing the PTMs is 8 (S67phosphorylation), 3 (K72dimethylation), and 2 (S66phosphorylation, K58dimethylation). In WT *D. melanogaster* gH1, K58 is on the *α*_1_-helix (Fig. 2 *C*) and has very limited contacts with the DNA in the docked encounter complexes. Thus, it is not surprising that dimethylation of K58 has only a modest effect on nucleosome binding. S66 points toward the LH core, which could explain the limited shifts in configuration observed upon S66phosphorylation (Fig. 2 *C*). On the other hand, S67 and K72 are both on the *α*_2_-helix (Fig. 2 *C*), and introduction of these PTMs at the interaction surface of the LH GD affects the LH binding pose and the number of H-bonds made by the neighboring residues (Fig. 6; Table S5).

Apart from S67phosphorylation, all PTMs resulted in an on-dyad binding mode to the most closed nucleosome conformation, snapshot 4, as observed for WT *D. melanogaster* gH1. Overall, though, for all 40 top-ranked docking encounter complexes for WT gH1 and gH1 with PTMs, 31 resulted in off-dyad binding to L-DNA and only 3 in off-dyad binding to L-DNA2.

For all four PTMs, the number and nature of the H-bonds with the nucleosome compared to WT *D. melanogaster* gH1 is affected for the two most populated encounter complexes (Table S8). For WT *D. melanogaster* gH1, the majority of the H-bonds with the nucleosome are made by K92 and K95 (6 H-bonds each) (Fig. 6; Table S5). For gH1 with PTMs, the most H-bonds are made by R63 (9 H-bonds, K72dimethylation, 6 H-bonds, S66phosphorylation), K91 (8 H-bonds, S67phosphorylation, 6 H-bonds, S66phosphorylation), and K107 (6 H-bonds, K72dimethylation) (Fig. 6; Table S5). Unlike the single-point mutants studied, the gH1 variants with PTMs bind differently to the most open nucleosome conformation, snapshot 8. This may be due to their greater size, which reduces steric accessibility to the N-DNA and results in encounter complexes further from the dyad axis. The high variation in hydrogen bonding upon introducing PTMs suggests that each posttranslationally modified LH could have unique nucleosome interaction features and thus may have a distinct regulatory effect on chromatin compaction and gene regulation.

## Conclusions

By BD docking of refined structures of LH GDs to nucleosome structures, we recapitulated the crystal structures of the complexes determined by Zhou et al. (7) and Bednar et al. (8). These results confirm that BD rigid-body docking is a valid tool for studying LH-nucleosome binding configurations and can be used without prior knowledge of the structural constraints on the structure of the complex. Our previous MD simulations suggested that both conformational selection and induced fit facilitate formation of the bound LH-nucleosome complex (54). Thus, it should be borne in mind that a complete understanding of chromatosome complexation by LH mutants will require further MD simulations to investigate the stability of the fully bound mutant complexes formed from the diffusional encounter complexes generated by BD docking.

The results of our BD docking simulations indicate that the chromatosome configuration is sensitive to single-point mutations and PTMs in the GD of LHs. We found that mutations changing the charge on *G. gallus* gH5 residues V80, K82, K85, and V87 and on *D. melanogaster* gH1 residues K102, I104, K107, and K109 around the LH *β*-turn significantly affect the LH configuration. The results show that both electrostatic and steric effects of the mutations and PTMs significantly influence the LH-nucleosome configuration. The computed LH GD-nucleosome interaction energies in the diffusional encounter complexes vary within a few kT in the different configurations. Thus, other mutations and PTMs on the nucleosome-binding faces of the LH GDs can be expected to affect LH-nucleosome configuration to varying extents.

Considering the diversity of species of the LHs used in recent experimental studies of LH-nucleosome complexes, our results indicate that a systematic comparison of chromatosome configurations for different LH and nucleosome sequences and single-point mutations is necessary to understand the distribution of the chromatosome structural ensemble and its effect on function. Moreover, experiments to investigate the structural ensemble in solution, such as hydroxyl radical footprinting or NMR, are important to complement crystallographic data. In higher eukaryotes, having a chromatosome structural ensemble could facilitate the ability of one LH isoform to substitute for other LH isoforms, for example, as indicated by recent experimental studies showing that a single LH isoform knockout is not lethal in mice (72).

Currently, there is a significant interest in determining the phenotypic effects of core histone tail PTMs. Here, we show that LH PTMs may alter the chromatosome structural ensemble, which may impact higher-order chromatin structure and possibly gene expression profiles. We found that S67phosphorylation and K72dimethylation cause the most significant shifts in chromatosome configuration, whereas S66phosphorylation and K58dimethylation have modest effects. Applying single-point mutations like K72R to prevent dimethylation (73) and S67E to partially mimic phosphorylation (74) of *D. melanogaster* gH1 could be a promising experimental approach to understand the phenotypic effects of these PTMs.

Our study has certain limitations that need to be borne in mind. First, in our BD simulations, rigid conformations of the molecules are used, and thus possible induced fit mechanisms that could further stabilize the LH-nucleosome complexes are neglected. Furthermore, the behavior of the mutants and PTMs of LHs used in our study could differ from the behavior in vivo because of the presence of the core histone and LH tails as well as the nucleosome connectivity via L-DNA in chromatin. Lastly, the nucleosome structure that we used in MD simulations to investigate off-dyad LH binding could have additional L-DNA conformations relevant to other LH binding modes that were not sampled.

In conclusion, by applying BD docking simulations, we find that the chromatosome structural ensemble is sensitive to specific LH mutations and PTMs, which may have implications for the effects of LH binding on chromatin structure and function.

## Author Contributions

M.A.Ö. carried out all calculations. All authors contributed to the design and analysis of the research and the writing of the manuscript.

## Supporting Material

Document S1. Figs. S1–S3 and Tables S1–S8

Document S2. Article plus Supporting Material
