# Supplementary material for: Dependence of Chromatosome Structure on Linker Histone Sequence and Posttranslational Modification
Source: Biophys J. 2018 May 11;114(10):2363–75. doi: 10.1016/j.bpj.2018.04.034 (PMC6129471; doi:10.1016/j.bpj.2018.04.034)
Supplement: Document S1. Figs. S1–S3 and Tables S1–S8 [file mmc1.pdf]

Biophysical Journal, Volume *114*

Supplemental Information

Dependence of Chromatosome Structure on Linker Histone Sequence and Posttranslational Modification

Mehmet Ali Öztürk, Vlad Cojocaru, and Rebecca C. Wade

Supplementary Figure 1: Structure based pairwise sequence alignments of the DNA in the nucleosomes used in this study. The systems studied are listed in Table 1 and referred to by the PDB identifier. The L-DNA1 and L-DNA2 linker DNA arms are labeled by bold and the nucleotides at the dyad point are highlighted in green. Pairwise alignments are shown as 4QLC and 5NL0 are very similar (51% sequence identity) while 1KX5/1ZBB is rather different in sequence (42% sequence identity to 4QLC and 31% to 5NL0).

```
4QLC        L-DNA2 ---------------ACTGGCCGCCCTGGAGAATCCCGGTGCCGAGGCCGCTCAATTGGT    45
5NL0        L-DNA2 ACTACGTAATATTGGCCAGCTAGGATATCACAATCCCGGTGCCGAGGCCGCTCAATTGGT    60
                                   * *   *      * *****************************

4QLC               CGTAGACAGCTCTAGCACCGCTTAAACGCACGTACGCGCTGTCCCCCGCGTTTTAACCGC   105
5NL0               CGTAGACAGCTCTAGCACCGCTTAAACGCACGTACGGAATCCGTACGTGCGTTTAAGCGG   120
                   ************************************   *     *     ***** **

4QLC               CAAGGGGATTACTCCCTAGTCTCCAGGCACGTGTCAGATATATACATCCTGTGCATGTAA   165
5NL0               TGCTAGAGCTGTCTACGACCAATTGAGCGGCCTCGGCACCGGGATTGTGATATCCTAGCT   180
                        *   *     * *        **                           *

4QLC               GT--------------- L-DNA1 167
5NL0               GGCCAATATTACGTAGT L-DNA1 197
                   *
```

```
4QLC        L-DNA2 ACTGGCCGCCCTGGAGAATCCCGGTGCCGAGGCCGCTCAATTGGTCGTAGACAGCTCTAG    60
1KX5/1ZBB   L-DNA2 ACTGGCCGCCCTGGAGAATCACCTGCAGATACTACCAAAAGTGTATTTGGAAACTGCTCC    60
                   ******************** *             *  ** **    * ** *   **

4QLC               CACCGCTTAAACGCACGTACGCGCTGTCCCCCGCGTTTTAACCGCCAAGGGGATTACTCC   120
1KX5/1ZBB          ATCAAAAGGCATGTTCAGCTGGAATCCAGCTGAACATGCCTTTTGATGGAGCAGTTTCCA   120
                     *         *  *    *   *    *      *           * * * *   *

4QLC               CTAGTCTCCAGGCACGTGTCAGATATATACATCCTGTGCATGTAAGT L-DNA1         167
1KX5/1ZBB          AATACACTTTTGGTAGTATCTGCAGGTTACATCCTGTGCATGTAAGT L-DNA1         167
                              *   ** ** *     ********************
```

```
5NL0       L-DNA2 ACTACGTAATATTGGCCAGCTAGGATATCACAATCCCGGTGCCGAGGCCGCTCAATTGGT    60
1KX5/1ZBB  L-DNA2 ---------------ACTGGCCGCCCTGGAGAATCACCTGCAGATACTACCAAAAGTGTA    60
                                  * *   *      * **** *             *  ** **

5NL0              CGTAGACAGCTCTAGCACCGCTTAAACGCACGTACGGAATCCGTACGTGCGTTTAAGCGG   120
1KX5/1ZBB         TTTGGAAACTGCTCCATCAAAAGGCATGTTCAGCTGGAATCCAGCTGAACATGCCTTTTG   120
                    * ** *   **    *       * *  *    *******    *  * *       *

5NL0              TGCTAGAGCTGTCTACGACCAATTGAGCGGCCTCGGCACCGGGATTGTGATATCCTAGCT   180
1KX5/1ZBB         ATGGAGCAGTTTCCAAATACACTTTTGGTAGTATCTGCAGGTTACATCCTGTGCATGTAA   167
                      **   * ** *    ** **  *             *  *         * *

5NL0              GGCCAATATTACGTAGT L-DNA1 197
1KX5/1ZBB         GT--------------- L-DNA1 167
                  *
```

Supplementary Figure 2: Calculation of L-DNA opening angles. **A-** The vectors v_L^1 and v_L^2 were defined based on selected DNA bases to represent the helical axes of L-DNA1 and L-DNA2, respectively, Öztürk et al. (3). The double headed arrows show the directionality of the L-DNA motions described by the two angles (γ_1 and γ_2). **B-** 8 Snapshots selected from a 100 ns standard MD simulation of the free nucleosome used for BD simulations (labeled in red) on the γ_1 and γ_2 histograms for L-DNA1 and L-DNA2, Öztürk et al. (3). Snapshots 1-4 have more closed conformations of the nucleosome and snapshots 6-8 have more open conformations of the nucleosome compared to snapshot 5, see Supplementary Figure 3. (Figure S2B is re-printed from Öztürk et al. (3) under Open access CC BY license.)

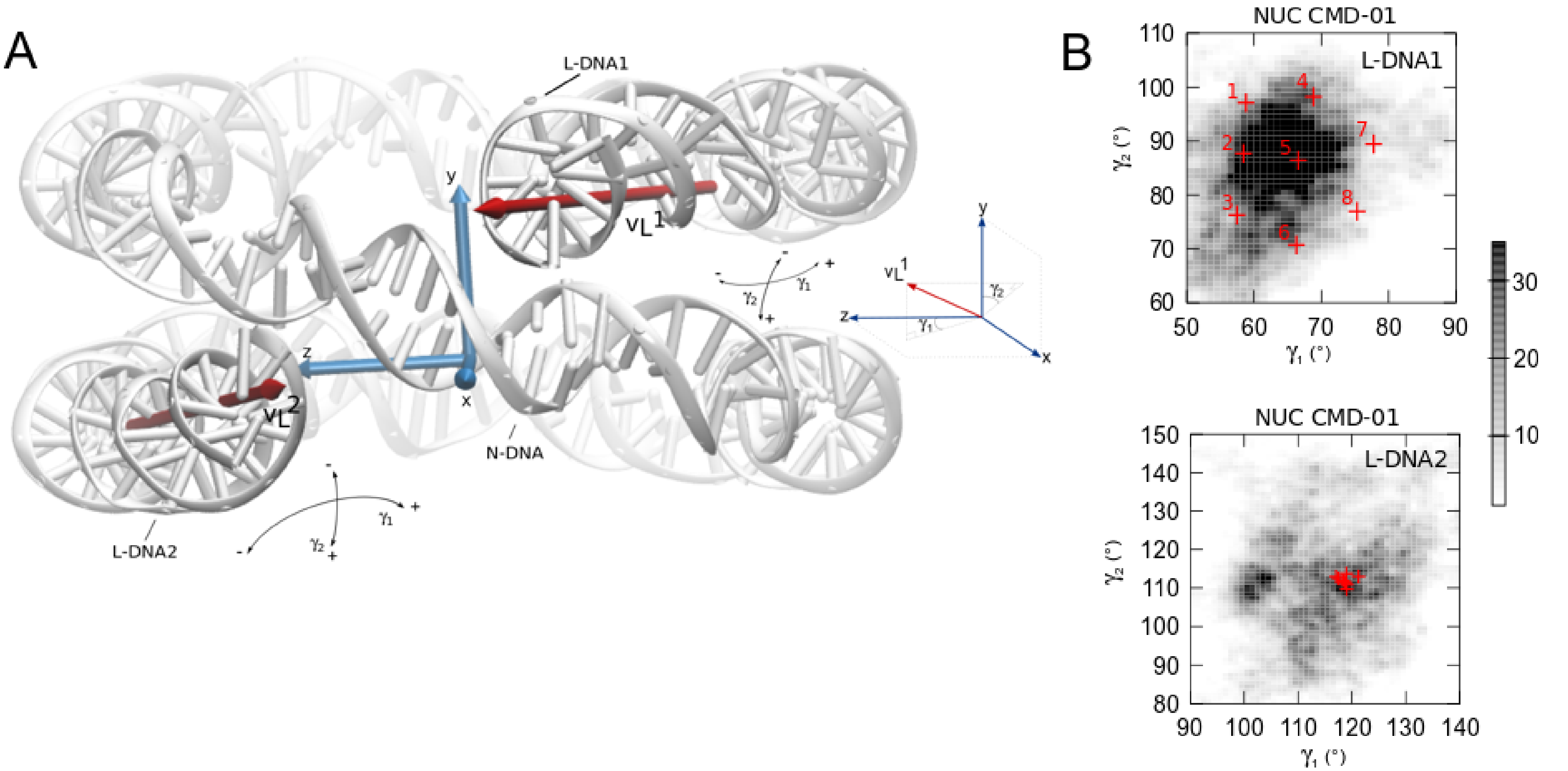

Supplementary Figure 3: Superposition of the 8 nucleosome snapshots selected from 100 ns standard MD simulation of the nucleosome for BD simulations (see Supplementary Figure 2). The DNA is colored according to snapshot and the core histones are shown in cartoon representation in gray. Snapshots 1-4 (1-blue, 2-green, 3-cyan, 4-lime) have more closed conformations of the nucleosome and snapshots 6-8 (6-pale pink, 7-red, 8-magenta) have more open conformations of the nucleosome compared to snapshot 5 (orange).

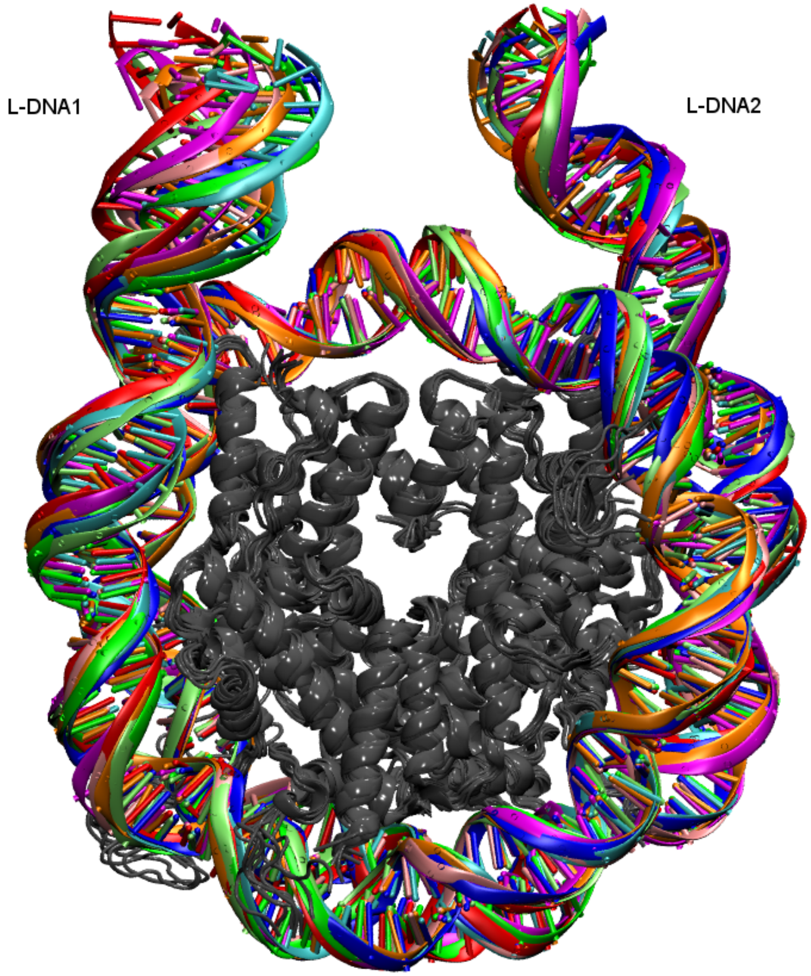

Supplementary Table 1 L-DNA opening angles are given for each L-DNA arm for the nucleosome structures used for the BD docking simulations with results given in Figure 5. The L-DNA1 arm is in a relatively closed form in nucleosome structures 1, 2, 3 and 4 and in a more open form for nucleosome structures 6, 7 and 8 (see Supplementary Figure 2 and reference (3) for further details.).

Nucleosome structure	L-DNA1 γ_1(°)	L-DNA1 γ_2(°)	L-DNA2 γ_1(°)	L-DNA2 γ_2(°)
**1**	58.7	97.0	119.0	109.7
**2**	58.4	87.6	118.3	111.8
**3**	57.4	76.2	118.7	111.5
**4**	68.8	98.2	119.0	110.9
**5**	66.5	86.4	121.2	112.9
**6**	67.2	77.2	118.6	110.0
**7**	77.7	89.4	117.0	112.9
**8**	75.2	76.9	118.9	113.7

The motions of the L-DNAs were described using the angles γ_1 and γ_2, where γ_1 = the angle between the xz projection of the vector v_L^1 or v_L^2 and the z axis, and γ_2 = the angle between the xy projection of v_L^1 or v_L^2 and the y axis. v_L^1 and v_L^2 were defined based on selected DNA residues along the helical axis of the two L-DNAs (See Supplementary Figure 2). v_L^1 connects the geometric centers of nucleotides 12-15, 320-323 and 2-5, 330-333, whereas v_L^2 connects the geometric centers of nucleotides 153-156, 179-182 and 163-166, 169-172. The numbering of the DNA nucleotides starts from 1 and 168 at the 5' ends of L-DNA1 and L-DNA2, respectively, and runs to 167 and 334 at the 3' ends of L-DNA2 and L-DNA1, respectively. All non-hydrogen atoms were used to define the nucleotides.

Supplementary Table 2 L-DNA opening angles are given for each L-DNA arm for the conformations derived from NMA using the nucleosome from the crystal structure with PDB id: 4QLC determined by Zhou et al. (1) and PDB id: 5NL0 determined by Bednar et al. (2). The conformations were generated along the first internal motion mode (mode 7, modes 1-6 correspond to rigid body translation and rotation). Mode 7_0 corresponds to the crystal structure. Mode 7_1 and mode 7_2 represent increasingly more open structures of the nucleosome. These three structures were used in BD docking to obtain the results given in Figure 4. See Supplementary Figure 2 for the definitions of the angles.

Nucleosome structure	L-DNA1 γ_1(°)	L-DNA1 γ_2(°)	L-DNA2 γ_1(°)	L-DNA2 γ_2(°)
**Crystal structure (4QLC) (Mode 7_0)**	56.0	98.9	111.2	78.3
**Mode 7_1**	57.1	99.8	105.7	77.4
**Mode 7_2**	58.5	101.9	99.9	76.2
**Crystal structure (5NL0) (Mode 7_0)**	60.5	84.5	108.7	83.7
**Mode 7_1**	62.4	85.2	105.6	82.5
**Mode 7_2**	64.1	85.9	102.6	81.3

The vectors for calculating the L-DNA angles were defined as described in Supplementary Table 1 using the corresponding aligned DNA bases.

Supplementary Table 3 BD docking simulations of LH binding to the nucleosome structures derived from the crystal structures with PDB ids: 4QLC (1) and 5NL0 (2). The orientations of the representative structures of the largest two clusters of encounter complexes obtained by docking WT *G. gallus* gH5 to the Zhou et al. (PDBid: 4QLC) nucleosome and WT *X. laevis* gH1 docking to the Bednar et al. nucleosome (PDB id: 5NL0) are given for docking to the nucleosome crystal structure (Mode 7_0) and two structures (Mode 7_1 and Mode 7_2) with slightly opened L-DNA arms.

Nucleosome structure (*)	gH5 WT					Nucleosome structure (*)	gH1 WT				
	N	%	α_3	β_1	l_1		N	%	α_3	β_1	l_1
Reference (4QLC)			-3 ↑	0	+3	Reference (5NL0)			-3 ↑	0	+3
Crystal structure (4QLC) (Mode 7_0)	2.0	35	-3 ↑	0	+3	Crystal structure (5NL0) (Mode 7_0)	1.5	39	+4 ↖	-4	-4
		31	+3 ↘	0, +3	-3			27	-3 ↑	0	+3
Mode 7_1	1.4	79	-3 ↑	0	+3	Mode 7_1	1.2	46	-4 →	-5	+5
		8	+1, +3 ↑	+1, +2	+2			34	-3 ↑	0	+3
Mode 7_2	2.0	36	-3 ↑	0, -3	0	Mode 7_2	1.0	85	-3 ↑	0	+3
		28	-3 ↑	0, -3	0			8	+3 ↓	0	-3

The DNA grooves on the nucleosome in contact with each structural element of LH (α_3, β_1 and l_1) are given in the respective columns (See Figure 3A). The arrows show the orientation of the LH α_3-helix when the nucleosome is aligned perpendicular to the viewing plane. N is the total number of encounter complexes that satisfy the docking criteria in each BD simulation, divided by 10^6. The % of these encounter complexes in the two largest BD clusters is given in the % column for clusters 1 (above) and 2 (below). (*) Normal Mode Analysis of an elastic network model of the nucleosome was performed. The crystal structure corresponds to mode 7_0 and modes 7_1 and 7_2 are structures along the slowest mode (mode 7) that have more open L-DNA arms than the crystal structure (See Methods and Supplementary Table 2 for details.)

Supplementary Table 4 The number of hydrogen bonds formed between nucleosomal DNA and wild-type and mutant *G. gallus* gH5 are summed over the 8 different *G. gallus* gH5 - nucleosome docking simulations, each using a different nucleosome conformation. Occurrences of 6 or more hydrogen bonds to a *G. gallus* gH5 residue are highlighted in red. Some residues have more than one hydrogen bond in the docked position.

**Residues**	**gH5 WT**	**V80K**	**K82I**	**K85V**	**V87K**
**S24**	0	0	1	0	0
**R37**	0	0	0	1	0
**R42**	3	3	3	2	0
**R47**	**8**	**9**	0	**10**	3
**Q48**	2	0	0	2	0
**K52**	0	**6**	0	0	0
**K55**	0	1	2	0	0
**K59**	0	0	2	0	0
**K69**	2	5	**8**	3	**12**
**R73**	0	2	2	3	1
**R74**	2	2	0	1	0
**A78**	0	1	0	0	0
**K82**	0	1	1	4	0
**K85**	2	1	2	3	2
**K87**	0	0	0	0	1
**S90**	0	1	0	0	1
**R94**	**14**	2	0	0	5
**K97**	2	4	0	2	2
**Total**	**35**	**38**	**21**	**31**	**27**

Supplementary Table 5 The number of hydrogen bonds formed between nucleosomal DNA and wild-type, mutant and post-translationally modified *D. melanogaster* gH1 summed over the 8 different *D. melanogaster* gH1 - nucleosome docking simulations, each using a different nucleosome conformation. Occurrences of 6 or more hydrogen bonds to a *D. melanogaster* gH1 residue are highlighted in red. Some residues have more than one hydrogen bond in the docked position.

Residues	gH1 WT	K102V	I104K	K107V	K109V	K58Dme	S66Phos	S67Phos	K72Dme
**K61**	0	1	0	0	0	0	0	0	0
**R63**	3	3	4	5	3	4	**6**	5	**9**
**S66**	0	0	1	0	0	0	0	0	1
**S67**	0	1	0	0	0	0	1	4	0
**L68**	0	0	0	2	0	0	0	0	0
**K72**	1	3	3	**6**	**7**	3	4	0	0
**K73**	3	3	0	2	0	2	1	5	0
**Q84**	0	0	1	0	0	0	0	0	0
**K85**	0	0	0	1	1	1	1	1	2
**K90**	0	0	0	0	0	0	1	0	0
**K91**	4	**6**	0	1	4	5	**6**	**8**	4
**K92**	**6**	3	1	4	2	2	1	1	1
**K95**	**6**	3	0	4	3	4	4	3	4
**I104**	0	0	1	0	0	0	0	0	0
**Q105**	0	0	0	0	0	0	1	0	0
**T106**	0	0	1	0	0	0	0	0	1
**K107**	3	2	4	0	3	**6**	3	5	**6**
**K109**	1	0	1	1	0	1	1	1	2
**A111**	0	0	0	1	0	0	0	0	0
**G113**	0	0	0	1	0	0	0	0	0
**K116**	0	0	1	0	0	0	0	0	0
**Total**	**27**	**25**	**18**	**28**	**23**	**28**	**30**	**30**	**30**

Supplementary Table 6 Docked configurations of WT and mutant *G. gallus* gH5 – nucleosome encounter complexes. The two largest encounter complexes are listed. Docking was performed to the 8 representative structures of the nucleosome from an MD simulation started from PDB id:1KX5. For details, see Table 1.

Snapshot	gH5B WT					gH5B V80K					gH5B K82I					gH5B K85V					gH5B V87K				
	N	%	α_3	β_1	l_1	N	%	α_3	β_1	l_1	N	%	α_3	β_1	l_1	N	%	α_3	β_1	l_1	N	%	α_3	β_1	l_1
1	2.7	68	+1 ↖	+1,+2	+2	3.8	46	+1 +2 X	+1,+2	+1	7.6	51	+3 ↓	0	-3	5.3	46	-3 ↑	0	+3	4.5	94	+3 ↓	0	-3
		29	-3 ↑	0	+2		35	+1 ↖	+1,+2	+2		9	+3 ↓	0	-3		23	0 ↙	0	+2		3	+3 ↓	0	-3
2	2.8	42	+3 ↓	0	-3	4.5	82	+1 ↑	+1,+2	+2	4.2	26	+3 ↓	0	-3	7.3	59	+1 ↖	+1,+2	+2	3.3	62	+1 ↖	+1,+2	+2
		42	+1 ↖	+1,+2	+2		8	+1, +2 ↙	+1,+2	+3		26	+3 ↓	0	-3		32	+1 ↖	+1,+2	+2		28	-2 ↘	-1,-2	-3
3	5.6	53	+1 ↖	+1,+2	+2	5.7	39	+2 X	+1,+2	no	2.1	59	+1 ↖	+1,+2	+2	6.4	38	+1 ↖	+1,+2	+2	5.4	95	+1 ↖	+1,+2	+2
		37	+1 ↖	+1,+2	+2		20	+2 X	+1,+2	no		28	0 ↓	0, -1	-3		25	+1 ↖	+1,+2	+2		2	-2 ↘	-1,-2	-3
4	3.0	67	+1 ↖	+1,+2	+2	2.1	39	-1 ↑	0	+3	4.1	40	0 ↓	0, -1	-3	3.9	48	+1 ↖	+1,+2	+2	3.9	48	+3 ↙	+3	+1
		17	0, +3 ↓	0, -1	no		28	+3 ↓	0	no		17	0 ↓	0, -1	-3		25	0 ↙	+1	+3		40	+1 ↖	+1,+2	+2
5	4.9	38	-1 ↘	-1,-2	-2	3.7	28	-1 ↘	-1,-2	-2	4.8	45	0 ↓	0, -1	-3	13.9	37	+1 ↖	+1,+2	+2	7.5	48	+1 ↖	+1,+2	+2
		24	-1 ↘	-1,-2	-2		21	+1, +2↙	+1,+2	no		14	0 ↓	0	-3		25	+1 ↖	+1,+2	+2		31	0 ↓	0	-3
6	3.3	58	+1 ↖	+1,+2	+2	5.4	61	+1↖	+1,+2	+2	1.6	85	-2 ←	-2	no	4.3	86	+1 ↖	+1,+2	+2	4.1	73	+1 ↖	+1,+2	+2
		29	+1 ↖	+1,+2	+2		16	+1↖	+1,+2	+2		6	+2 ←	+2	no		6	+1,+2 ↖	+1,+2	no		15	0 ↑	0	+2
7	7.4	27	+1 ↖	+1,+2	+2	9.4	41	-1 ↘	-1,-2	-2	0.7	42	+1↖	+1	+2	8.1	26	+1 ↖	+1,+2	+2	3.1	96	+1 ↖	+1,+2	+2
		20	+1 ↖	+1,+2	+2		37	-1 ↘	-1,-2	-2		31	-1 ↓	0	no		24	+1 ↖	+1,+2	+2		2	-1 ↘	-1,-2	-2
8	5.0	42	-1 ↘	-1,-2	-2	5.6	74	-1 ↘	-1,-2	-2	2.6	33	-1 ↘	-1,-2	-2	6.1	44	+1 ↖	+1,+2	+2	5.0	42	-1 ↘	-1,-2	-2
		28	-1 ↘	-1,-2	-2		10	-1 ↙	-1	-2		23	-1 ↘	-1,-2	-2		16	-1 ↘	-1,-2	-2		28	-1 ↘	-1,-2	-2

Supplementary Table 7 Docked configurations of WT and mutant *D. melanogaster* gH1 – nucleosome encounter complexes. The two largest encounter complexes are listed. Docking was performed to 8 representative structures of the nucleosome from an MD simulation started from PDB id:1KX5. For details, see Table 1.

Snapshot	gH1 WT					gH1 K102V					gH1 I104K					gH1 K107V					gH1 K109V				
	N	%	α_3	β_1	l_1	N	%	α_3	β_1	l_1	N	%	α_3	β_1	l_1	N	%	α_3	β_1	l_1	N	%	α_3	β_1	l_1
1	2.3	25	+1↖	+1, +2	+2	1.9	50	+1↖	+1,+2	+2	2.4	66	+1↖	+1,+2	+2	1.1	39	+1↖	+1,+2	+2	1.6	47	0 ↑	0	+2
		21	-3 ↖	-3	no		21	0 ↓	-1,-3	-3		9	+1↖	+1,+2	+2		25	0, -1 ↓	-1,-2	no		28	0 ↑	0	+2
2	3.3	97	-3 ←	-2	no	1.8	49	-2 ↗	-1,-2	-3	3.9	72	+1↖	+1,+2	+2	1.2	57	-3 ↙	-2	no	1.8	70	+1↖	+1,+2	+2
		2	-1 ↙	-1,-2	-3		20	+1↖	+1,+2	+2		15	-2 →	-1,-2	-2		34	-2 ↗	-3	-3		15	-2 ↗	-1, -2	-3
3	3.9	60	-3 ←	-2	-1	2.7	27	-1 ↙	-1,-2	-2	4.4	65	+1,+2 ↓	+1,+2	0	1.7	55	-2 ↗	-3	-3	2.2	54	-1, -2 ↗	-2	-3
		31	-1 ↙	-1,-2	-2		26	-1, -2 ↖	-1,-2	0		21	-1 ↘	-1,-2	-2		22	0, -1 ↙	-1	-3		23	+2 ↓	+1,+2	no
4	2.7	83	0 ↖	+3	+3	1.3	47	+2 →	+2	no	3.1	92	+3 ↙	0,+3	0	0.9	42	+3 ↙	0	no	0.9	69	+3 ↙	0,+3	0
		4	-3 ↖	-2	-1		20	-3 ↖	-1	+3		6	+1↖	+1,+2	+2		25	-3 ↗	-1	no		14	-1 ↗	0	+3
5	4.0	59	-1, -2 ↗	-1, -2	-3	4.9	36	-1, -2 ↗	-1,-2	-3	4.5	51	+1↖	+1,+2	+2	3.5	22	-1, -2 ↗	-1,-2	-3	1.8	53	+1↖	+1,+2	+2
		27	-2 ↖	-2	no		28	-1, -2 ↗	-1,-2	-3		35	-1, -2 →	-1,-2	-2		21	-1, -2 ↗	-1,-2	-3		28	-1, -2 ↗	-1,-2	-3
6	1.9	63	-2 ←	-2	no	2.1	55	-2 ↗	-1,-2	-3	5.3	95	+1↖	+1,+2	+2	3.9	55	-2 ←	-2	no	2.3	32	-1, -2 ↗	-1,-2	-3
		29	-1,-2 ↗	-1,-2	-3		13	-1, -2 ↗	-1,-2	-3		1	-1 ↘	-1,-2	-2		24	-1, -2 ↗	-1,-2	-3		32	+1↖	+1,+2	+2
7	1.5	49	-2 ←	-2	no	1.9	74	-2 ←	-2	no	5.8	27	-1 ↘	-1,-2	-2	2.6	44	-2 ←	-2	no	1.9	78	-1 ↘	-1,-2	-2
		22	-3 ↖	-2	-1		14	+1↖	+1,+2	+2		24	-1 ↘	-1,-2	-2		30	-2 ←	-2	no		12	-1 ↘	-1,-2	-2
8	4.0	75	-1 ↘	-1,-2	-2	2.2	64	+1↖	+1,+2	+2	3.6	61	-1 ↘	-1,-2	-2	3.3	29	-2 ←	-2	no	1.8	77	-1 ↘	-1,-2	-2
		8	-1 ↘	-1,-2	-2		19	-1 ↘	-1,-2	-2		28	-1 ↘	-1,-2	-2		28	-1, -2 ↗	-1,-2	-3		9	-1,-2 ↘	-1,-2	-3

Supplementary Table 8 Docking configurations of WT and post-translationally modified *D. melanogaster* gH1 – nucleosome encounter complexes. The two largest encounter complexes are listed. Docking was performed to 8 representative structures of the nucleosome from an MD simulation started from PDB id:1KX5. For details, see Table 1.

Snapshot	gH1 WT					gH1 K58 dimethylation					gH1 S66 phosphorylation					gH1 S67 phosphorylation					gH1 K72 dimethylation				
	N	%	α_3	β_1	l_1	N	%	α_3	β_1	l_1	N	%	α_3	β_1	l_1	N	%	α_3	β_1	l_1	N	%	α_3	β_1	l_1
1	2.3	25	+1 ↖	+1, +2	+2	2.3	27	-3 ←	-2	no	1.9	35	-3 ←	-2	no	1.4	57	-3 ←	-2	no	2.2	47	0 ↓	0, -1	-3
		21	-3 ↖	-3	no		19	0 ↓	0, -1	-3		14	0 ↓	0, -3	no		13	0 ↙	0, -1	-3		33	0, -1 ↙	-1, -3	-3
2	3.3	97	-3 ←	-2	no	3.7	90	-3 ←	-2	no	3.4	97	-3 ←	-2	no	3.0	43	-3 ←	-2	-1	3.8	96	-3 ←	-2	no
		2	-1 ↙	-1,-2	-3		5	-3 ←	-2	no		1	-3 ←	-2	no		24	-3 ←	-2	-1		3	0 ↓	0, -1	-3
3	3.9	60	-3 ←	-2	-1	3.4	65	-3 ←	-2	-1	2.8	47	-3 ←	-2	-1	2.0	36	-1 ↘	-1, -2	-2	3.9	82	-3 ←	-2	-1
		31	-1 ↙	-1,-2	-2		19	-1 ↘	-1, -2	-2		40	-1 ↘	-1, -2	-2		22	-2 ↖	-2	-1		7	-1, -2 ↓	-1, -2	-2
4	2.7	83	0 ↖	+3	+3	2.3	73	0 ↖	+3	+3	1.8	67	0 ↑	+3	+3	1.6	30	+3 ↙	+3	+1	2.6	70	0 ↖	+3	+3
		4	-3 ↖	-2	-1		6	-3 ↖	-1, -2	-1		13	-3 ↙	0, -3	0		29	+3 ↙	+3	+1		11	0 ↑	+3	+3
5	4.0	59	-1, -2 ↗	-1, -2	-3	3.1	60	-1,-2 ↗	-1,-2	-3	2.7	50	-1,-2 ↗	-1,-2	-3	2.2	59	-2 ←	-2	no	2.3	36	-1,-2 ↗	-1, -2	-3
		27	-2 ↖	-2	no		23	-2 ←	-2	no		30	-2 ←	-2	no		25	+1, +2 ↓	+1,+2	0		20	-1,-2 ↗	-1, -2	-3
6	1.9	63	-2 ←	-2	no	1.7	71	-2 ←	-2	no	1.5	72	-2 ←	-2	no	1.1	33	-1,-2 ↗	-1, -2	-3	2.0	35	-2 ←	-2	no
		29	-1,-2 ↗	-1,-2	-3		20	-1,-2 ↗	-1, -2	-3		16	-1,-2 ↗	-1, -2	-3		23	+2, +3 ↙	0, +3	0, +3		18	-3 ↖	-1, -2	-1
7	1.5	49	-2 ←	-2	no	1.7	55	-2 ←	-2	no	1.4	44	-2 ←	-2	no	1.1	45	-1 ↘	-1, -2	-2	1.9	46	-3 ↖	-2	-1
		22	-3 ↖	-2	-1		25	-3 ↖	-2	-1		36	-3 ↖	-2	-1		34	-3 ↖	-2	-1		39	-3 ↖	-2	-1
8	4.0	75	-1 ↘	-1,-2	-2	3.9	59	-2 ↖	-2	no	3.7	50	-2 ↖	-2	no	4.0	26	+3 ↙	0, +3	+1	3.2	47	-2 ↖	-2	no
		8	-1 ↘	-1,-2	-2		24	-2 ↖	-2	no		21	-2 ↖	-2	no		21	+3 ↙	0, +3	+1		11	-1 ↘	-1, -2	-2

References:

1. Zhou, B.-R., J. Jiang, H. Feng, R. Ghirlando, T.S. Xiao, and Y. Bai. 2015. Structural Mechanisms of Nucleosome Recognition by Linker Histones. Mol. Cell. 59: 628–638.

2. Bednar, J., I. Garcia-Saez, R. Boopathi, A.R. Cutter, G. Papai, A. Reymer, S.H. Syed, I.N. Lone, O. Tonchev, C. Crucifix, H. Menoni, C. Papin, D.A. Skoufias, H. Kurumizaka, R. Lavery, A. Hamiche, J.J. Hayes, P. Schultz, D. Angelov, C. Petosa, and S. Dimitrov. 2017. Structure and Dynamics of a 197 bp Nucleosome in Complex with Linker Histone H1. Mol. Cell. 66: 384–397.e8.

3. Öztürk, M.A., G.V. Pachov, R.C. Wade, and V. Cojocaru. 2016. Conformational selection and dynamic adaptation upon linker histone binding to the nucleosome. Nucleic Acids Res. 44: 6599–6613.
