# Supplementary material for: Dependence of Chromatosome Structure on Linker Histone Sequence and Posttranslational Modification
Source: Biophys J. 2018 May 11;114(10):2363–75. doi: 10.1016/j.bpj.2018.04.034 (PMC6129471; doi:10.1016/j.bpj.2018.04.034)
Supplement: Document S2. Article plus Supporting Material [file mmc2.pdf]

Biophysical Journal
Article

Biophysical Society

Dependence of Chromatosome Structure on Linker Histone Sequence and Posttranslational Modification

Mehmet Ali Öztürk,[1,2] Vlad Cojocaru,[3,4] and Rebecca C. Wade[1,5,6,*]

[1]Molecular and Cellular Modeling Group, Heidelberg Institute for Theoretical Studies (HITS), Heidelberg, Germany; [2]The Hartmut Hoffmann-Berling International Graduate School of Molecular and Cellular Biology, Heidelberg University, Heidelberg, Germany; [3]Computational Structural Biology Laboratory, Department of Cellular and Developmental Biology, Max Planck Institute for Molecular Biomedicine, Münster, Germany; [4]Center for Multiscale Theory and Computation, Westfälische Wilhelms University, Münster, Germany; [5]Center for Molecular Biology (ZMBH), DKFZ-ZMBH Alliance, Heidelberg University, Heidelberg, Germany; and [6]Interdisciplinary Center for Scientific Computing (IWR), Heidelberg, Germany

ABSTRACT Linker histone (LH) proteins play a key role in higher-order structuring of chromatin for the packing of DNA in eukaryotic cells and in the regulation of genomic function. The common fruit fly (*Drosophila melanogaster*) has a single somatic isoform of the LH (H1). It is thus a useful model organism for investigating the effects of the LH on nucleosome compaction and the structure of the chromatosome, the complex formed by binding of an LH to a nucleosome. The structural and mechanistic details of how LH proteins bind to nucleosomes are debated. Here, we apply Brownian dynamics simulations to compare the nucleosome binding of the globular domain of *D. melanogaster* H1 (gH1) and the corresponding chicken (*Gallus gallus*) LH isoform, gH5, to identify residues in the LH that critically affect the structure of the chromatosome. Moreover, we investigate the effects of posttranslational modifications on the gH1 binding mode. We find that certain single-point mutations and posttranslational modifications of the LH proteins can significantly affect chromatosome structure. These findings indicate that even subtle differences in LH sequence can significantly shift the chromatosome structural ensemble and thus have implications for chromatin structure and transcriptional regulation.

INTRODUCTION

Olins and Olins reported the first electron micrograph of the beads-on-a-string structure of chromatin in 1974 (1). Shortly afterwards, on the basis of biochemical and crystallographic data, Kornberg formulated the nucleosome hypothesis, namely, that in eukaryotes, chromatin consists of repeating units of ~200 bp DNA wrapped around core histone oligomers connected to form a flexibly jointed chain (2), and this was supported by further electron microscopy evidence (3). Digestion of chromatin by a nonspecific nuclease revealed subnucleosomal particles, chromatosomes, connected by linker DNA (4). Each chromatosome consists of a nucleosome core particle of 147 bp of nucleosomal DNA (N-DNA) coiled around a core histone octamer extended by ~20 bp of linker DNA (L-DNA) and bound by one linker histone (LH). Thus, the chromatosome can be considered as a fundamental unit of chromatin structure (5). Structurally, LHs are composed of ~200 amino acid (aa) residues and have three domains: an ~40 aa unstructured N-terminal tail, a conserved ~80 aa globular domain (GD), and an ~100 aa disordered C-terminal tail. Because of the flexibility of the N- and C-terminal tails, only the GD has been crystallized and its structure determined by x-ray crystallography (*Gallus gallus* gH5, Protein Data Bank (PDB): 1HST, 2.6 Å resolution) (6). Despite the recent determination of the crystal structures of LH GD-nucleosome complexes (PDB: 4QLC, 3.5 Å resolution (7); PDB: 5NL0, 5.4 Å resolution (8)), the structural determinants of chromatosome formation are still not well understood. In two studies by Zhou et al. (7,9), the authors reported that the *G. gallus* LH isoform (gH5) binds on-dyad to a nucleosome with a Widom 601 DNA sequence, whereas the *Drosophila melanogaster* LH globular domain H1 (gH1) binds off-dyad to the same nucleosome. Interestingly, in a follow-up study, by using low-resolution spin-labeling experimental constraints, Zhou et al. (10) suggested that the on-dyad binding mode of the *G. gallus* gH5 to the nucleosome could be switched to an off-dyad binding mode by introducing a pentamutation in

Submitted January 2, 2018, and accepted for publication April 9, 2018.

*Correspondence: rebecca.wade@h-its.org

Editor: Tamar Schlick.

https://doi.org/10.1016/j.bpj.2018.04.034

© 2018 Biophysical Society.

This is an open access article under the CC BY license (http://creativecommons.org/licenses/by/4.0/).

the *G. gallus* gH5. These results suggest that it is important to understand the sequence dependence of the structure of the chromatosome, which can have different LH variants and nucleosome sequence combinations.

Various experiments suggest specific effects of LH variants on DNA binding and chromatin condensation. Orrego et al. reported up to 19-fold differences in affinity to chromatin for LH H1 variants (11), and Clausell et al. obtained similar results from atomic force microscopy (12). Brown and colleagues used mutagenesis and fluorescence recovery after photobleaching to map the regions affecting chromatin-binding affinity in H1.1–H1.5 and to identify distinct nucleosome binding surfaces in H1c and H1(0) (13,14). It was also found that individual LH variants can trigger apoptosis (15) and are differentially expressed during stem cell differentiation, cell cycle progression, and proliferation (16,17). The specificities and genomic distribution of LH variants was recently reviewed by Kowalski and Palyga (18) and Millán-Ariño et al. (19). These data suggest that LH variants may have distinct functions because of different nucleosome interaction and chromatin compaction mechanisms.

The first posttranslational modification (PTM) of an LH was reported in 1972 (20). Since then, many studies have shown that LHs can have methylation, acetylation, ADPribosylation, ubiquitination, formylation, and PARylation PTMs (21–32). Izzo and Schneider recently extensively reviewed human and mouse H1 PTMs (33). They reported that H1 phosphorylation can have opposing effects on chromatin condensation. Horn et al. suggested that H1 phosphorylation may regulate ATP-dependent chromatin remodeling enzymes and thus impact chromatin compaction (34). Furthermore, high H1 phosphorylation levels are linked with DNA repair (35), apoptosis (36), cellular aging (37), and cancer events (38). H1 methylation is also associated with heterochromatin organization (39) and cell-cycle-regulated chromatin binding (26). However, although many sites of variant specific PTMs have been characterized, the phenotypic impact of individual LH PTMs is often unknown (40).

A range of computational approaches has been used to model and simulate LH-nucleosome complexes. Mesoscale simulations have been applied to explore the influence of LH concentration, conformation, and nucleosome interactions on chromatin structure as well as the dependence of LH-chromatin interactions on salt concentration (41–45). Most approaches to obtain atomic-detail structures of LH-nucleosome complexes have employed computational docking subject to experimental constraints (46–48). Most recently, Zhou et al. (9,10) used HADDOCK (49) and Bednar et al. (8) used Autodock Vina (50) to determine structures of LH GD-nucleosome complexes based on experimental constraints. We have previously shown that Brownian dynamics (BD) rigid-body docking can be used for electrostatically driven macromolecular docking to generate diffusional encounter complexes (51,52) and could be used without experimental constraints to generate structures of *G. gallus* gH5-nucleosome encounter complexes that were consistent with the available experimental data (53). We then performed atomic-detail molecular dynamics (MD) simulations starting from the BD encounter complexes, which, by taking LH GD and nucleosome flexibility into account, revealed a binding mechanism involving conformational selection and induced fit (54). In the bound complex with an off-dyad position of *G. gallus* gH5, we found that the gH5 β_1-loop V78 makes hydrophobic contacts with the DNA and stabilizes the complex (54). There are exchanges of positive with hydrophobic residues at three positions in the β_1-loop of the LH between *G. gallus* gH5 and *D. melanogaster* gH1 sequences (Figs. 1 and 2), suggesting that mutants with single-point mutations on the β_1-loop could help to understand the determinants of chromatosome structure.

Although atomic-detail and coarse-grained MD simulations have been applied to study the effects of PTMs of core histone tails on protein binding (41,55), on nucleosome structure (56), and on internucleosome interactions (41), no such studies have yet been reported for variants or PTMs of LHs. In this study, we apply the BD docking approach to investigate the effects of sequence variation and PTMs on the binding configurations of *G. gallus* gH5 and *D. melanogaster* gH1 to the nucleosome. The computational efficiency of the BD approach allows us to consider a number of mutations and PTMs. Moreover, docking is performed for different nucleosome conformations, allowing the relation between LH binding mode and nucleosome opening to be explored. The disordered N- and C-terminal domains of the LH are not included in the models, as it has been shown that they do

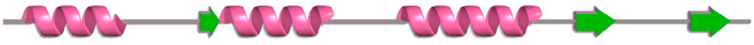

```
SP|P02259|H5_CHICKEN     22   SHPTYSEMIAAAIRAEKSRGGSSRQSIQKYIKSHYKVGHNA-DLQIKLSIRRLLAAGVLKQTKGVGASGSFRLAK    97
SP|P02255|H1_DROSOPHILA  45   SHPPTQQMIDAAIKNLKERGGSSLLAIKKYITATYKVDAQKLAPFIKKYLKSLVVNGKLIQTKGKGASGSFKLS-   118
SP|P22844|H1_XENOPUS     25   -HPKYSDMILAAVQAEKSRSGSSRQSIQKYIKNHYKVGENA-DSQIKLSIKRLVTSGALKQTKGVGASGSFRLAK    97
                               **   .:** **::   *.*.***   :*:***.   ***. :      **   :: *:.  * * **** ******:*:
```

FIGURE 1 Sequence alignment of the globular domains (GD) of the *G. gallus* H5, *D. melanogaster* H1, and *X. laevis* H1 isoforms. The three LH GD structures have 45% sequence identity. The secondary structure of the GDs is shown above the alignment. Uniprot accession numbers are given at the beginning of each row. Residues that are mutated in *G. gallus* gH5 and *D. melanogaster* gH1 in this work are shown in red. Residues that are posttranslationally modified in *D. melanogaster* gH1 are shown in blue (see Fig. 2). Note that Zhou et al. used a *D. melanogaster* gH1 construct that has core-stabilizing mutations at the residues shown in magenta (9). For consistency, we used the same construct in our simulations for our reference WT *D. melanogaster* gH1. To see this figure in color, go online.

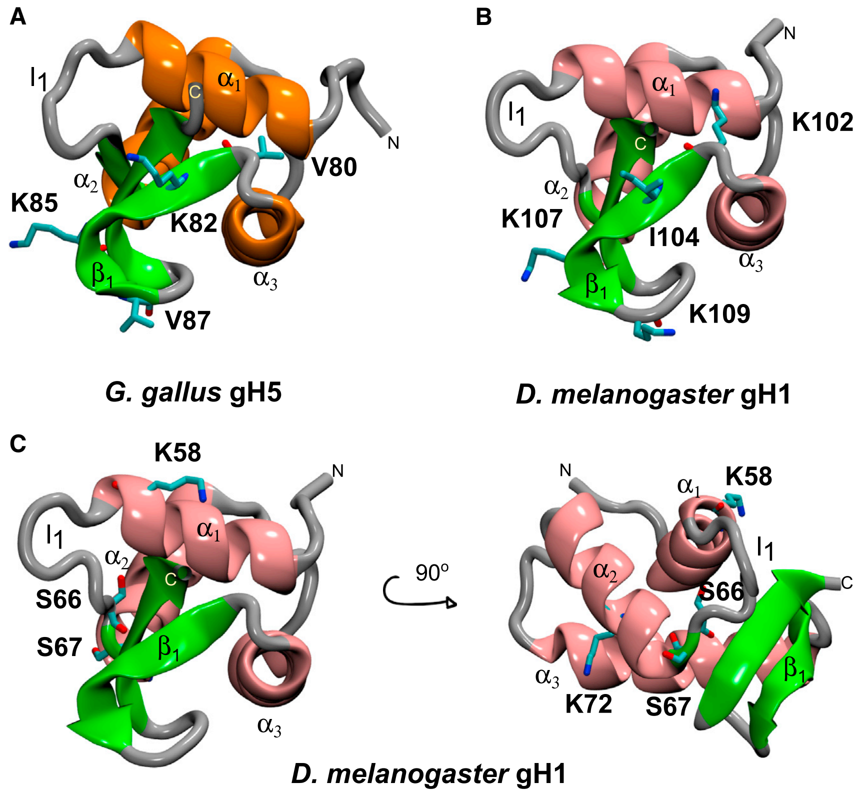

FIGURE 2 Structures of the LH GDs studied. (*A*) The *G. gallus* gH5 structure, showing the positions of the mutated residues V80, K82, K85, and V87, is given. (*B*) The *D. melanogaster* gH1 structure, showing the positions of the mutated residues K102, I104, K107, and K109, is given. (*C*) The *D. melanogaster* gH1 structure in two orientations, showing the sites of PTMs K72dimethylation, S67phosphorylation, S66phosphorylation, and K58dimethylation, is given. The LH GDs are shown in cartoon representation and colored according to secondary structure: α-helices in orange or pink, β-sheets in green, and unstructured regions in gray. Mutated side chains are shown in stick representation with coloring by atom type. To see this figure in color, go online.

not affect the location of the GD LH on the nucleosome (7,8), although the C-terminus affects the affinity (57).

We first validated our BD docking protocol by testing its reproduction of crystallographic structures of LH-nucleosome complexes. We then introduced single-point mutations into both LHs, and by docking the mutants to nucleosome structures, we identified residues that switch chromatosome configurations. Furthermore, we analyzed the effects of *D. melanogaster* gH1 PTMs on LH-nucleosome binding and the distribution of the chromatosome structural ensemble.

MATERIALS AND METHODS

We prepared five sets of systems for BD docking simulations (see Table 1). Each system consisted of an LH GD structure and a nucleosome structure to which the LH GD was docked.

LH GD-nucleosome structures

Recently, two crystal structures were reported for LH GD-nucleosome complexes (PDB: 4QLC (7) and 5NL0 (8)). To confirm the validity of our computational protocol, we used these structures as control test systems. From each structure, we created two PDB files, one for the nucleosome and one for the LH GD. Conformational variability of the nucleosome was considered as done previously (53) by generating a set of structures by performing an elastic network normal mode analysis (NMA) using the NOMAD-Ref server (58). For the nucleosome structure from PDB: 5NL0, the following parameters were used so as to generate structures with slightly more open L-DNA arms than the crystal structure: number of modes to calculate, 106; distance weight parameter for elastic constant, 5 Å; elastic network model cutoff for mode calculation, 10 Å; average root mean-square deviation (RMSD) in output trajectories from the initial structure, 3 Å; and calculation method, all-atom and automatic. For the nucleosome from PDB: 4QLC, we used the same nucleosome structures obtained with the default NOMAD-Ref server parameters as used in our previous study (54), which were number of modes to calculate, 16; distance weight parameter for elastic constant, 5 Å; elastic network model cutoff for mode calculation, 10 Å; average RMSD in output trajectories from the initial structure, 1 Å; and calculation method, all-atom and automatic. The output structures of the nucleosomes were named mode 7_0 (crystal structure), mode 7_1, and mode 7_2 and correspond to snapshots along the lowest frequency mode (mode 7, modes 1–6 correspond to rigid body translation and rotation) with increasingly more open L-DNA arms.

As homology-modeled LH structures were used to fit the LH densities in the recent crystal structures, the LH structures extracted from these PDB files were refined using the GalaxyRefine web server tool (59) to increase the structural quality of the side chains of the LHs by using the “mild relaxation only” option. The GalaxyRefine tool rebuilds side chains and performs side-chain repacking and structure relaxation with an MD-simulation-based protocol. The tool ranked best for improving the local structure quality in the CASP10 assessment (59). In all refinements, the all-atom RMSD of the input and output LH structures of the GalaxyRefine tool were below 2 Å.

Apo-nucleosome structures

The eight snapshots from the MD simulation of an apo-nucleosome performed by Öztürk et al. (54) and previously used for BD rigid-body docking simulations were used. Öztürk et al. showed that the BD docking to these snapshots resulted in similar LH binding configurations to those obtained for nucleosome structures generated by NMA by Pachov et al. (53). Here, we used our previously generated snapshots of nucleosome structures from MD simulations rather than NMA, as MD provides more realistic structures

TABLE 1 Systems Used in BD Docking Simulations

Nucleosome Structures	DNA Sequence	Core Histones	Number of Nucleosome Conformations for Docking	L-DNA Length (bp)	LH Globular Domain with Conformation in Parentheses	BD Simulations
Crystal structure (PDB: 4QLC, Zhou et al. (7)) and structures from NMA	Widom 601	*D. melanogaster*	3	10	*G. gallus* gH5 (closed)	protocol validation (Fig. 4 *A*)
Crystal structure (PDB: 5NL0, Bednar et al. (8)) and structures from NMA	Widom 601L	*X. laevis*	3	26	*X. laevis* gH1 (closed)	protocol validation (Fig. 4 *B*)
MD snapshots (Öztürk et al. (54) based on PDB: 1KX5 and 1ZBB	palindromic *H. sapiens* X chromosome α-satellite sequence	*X. laevis*	8	10	*G. gallus* gH5 (closed)	gH5 mutants: V80K K82I K85V V87K (Fig. 5 *A*)
MD snapshots (Öztürk et al. (54) based on PDB: 1KX5 and 1ZBB	palindromic *H. sapiens* X chromosome α-satellite sequence	*X. laevis*	8	10	*D. melanogaster* gH1 (same as Zhou et al. (9))	gH1 mutants: K102V I104K K107 K109V (Fig. 5 *B*)
MD snapshots (Öztürk et al. (54) based on PDB: 1KX5 and 1ZBB	palindromic *H. sapiens* X chromosome α-satellite sequence	*X. laevis*	8	10	*D. melanogaster* gH1 (same as Zhou et al. (9))	gH1 PTMs: K58 dimethylation S66 phosphorylation S67 phosphorylation K72 dimethylation (Fig. 5 *C*)

The five different simulation systems and the details of their structural components are given. See Fig. S1 for a comparison of the three different DNA sequences in the nucleosomes studied.

than NMA, albeit at greater computational cost. These nucleosome structures were derived from the crystal structures with PDB: 1KX5 (1.9 Å resolution) (60) for the nucleosome core particle and with PDB: 1ZBB (9 Å resolution) (61) for the 10 bp extensions of each L-DNA. The following procedure was previously used (54): the N-DNA was extended with two L-DNA arms and core histone tails were removed. Nucleosome dynamics were simulated for 100 ns by standard MD simulation. After clustering of structures from the trajectory, eight different snapshots were selected to cover the conformational space of the nucleosome, in which the L-DNA2 arm was in a highly populated conformation and the conformation of the L-DNA1 arm varied (for details of the nucleosome structures, see Figs. S2 and S3; Table 1; (54)). The eight nucleosome structures have different L-DNA1 arm opening and closing angles: snapshots 6, 7, and 8 have a more open L-DNA1 arm, and snapshots 1, 2, 3, and 4 have a more closed L-DNA1 arm compared to snapshot 5 (see Figs. S1–S3; Table 1).

LH GD structure

The refined *G. gallus* gH5 crystal structure (PDB: 1HST, chain B (6)) was used for docking to the apo-nucleosome structures. Exchanges of positive and hydrophobic residues at four positions in the β_1-loop of the *G. gallus* gH5 and *D. melanogaster* gH1 sequences were selected (Figs. 1 and 2 *A*). The V80K, K82I, K85V, and V87K mutations were introduced individually into *G. gallus* gH5 to construct single-point mutants. The structure of *D. melanogaster* gH1 as reported by Zhou et al. (2013) (9) was kindly provided by Yawen Bai, and the K102V, I104K, K107V, and K109V mutations were introduced into *D. melanogaster* gH1. All mutations were introduced using the PyMOL molecular modeling software (62) (Figs. 1 and 2, *A* and *B*), and then each structure was refined using the GalaxyRefine structure refinement web server tool (59) as described above.

Additionally, the K72dimethylation, S67phosphorylation, S66phosphorylation, and K58dimethylation PTMs (Fig. 2 *C*) (26) were introduced into the *D. melanogaster* gH1 by applying the PyTMs plugin in PyMOL (62,63) to the refined wild-type (WT) structure. As the GalaxyRefine web server only accepts standard amino acids, partial atomic charges and radii of the posttranslationally modified residues were obtained from previously published studies (64,65) and added manually to the PQR files generated for these structures without further refinement.

BD preparation and simulation parameters

For BD simulations, polar hydrogen atoms were added to the structures by using the PDB2PQR 2.1.1 web-server (66), and partial atomic charges and atomic radii were assigned by using the AMBER99 force field (67). For all structures, the molecular electrostatic potentials were calculated by using APBS 1.4 (68) to solve the nonlinear Poisson-Boltzmann equation with a

1 Å grid spacing. Input parameters were a temperature of 298.15 K, solvent- and solute-relative dielectric constants of 78.54 and 2, respectively, and an ionic strength of 100 mM. The van der Waals surface was used to define the dielectric boundary. Effective charges were assigned to charged residues on the protein and to P atoms on the DNA using the Effective Charges for Macromolecules (ECM) program (69). BD simulations were performed by using the SDA 7 software (70) with electrostatic interaction forces and neglecting short-range interactions. The solutes diffused as rigid bodies and overlap between the GD LH and the nucleosome was prevented by applying a 0.5 Å excluded volume criterion. The BD trajectories were started with the geometric center of LH GD positioned randomly on a sphere centered on the nucleosome at a center-to-center distance of ~185 Å and stopped at a center-to-center distance of ~204 Å. A time step of 1 ps was used. For each system, we generated 20,000 BD trajectories, and our test runs for 10,000 BD trajectories and for different initial random-number seeds resulted in similar cluster configurations and population percentages. The following two geometric conditions were used to define formation of the diffusional encounter complexes: 1) the geometric center-to-center distance of LH and the nucleosome <98 Å, and 2) the nucleosome dyad point and LH center separation <40 Å. The coordinates and interaction energies of a complex at a given time step were recorded if the RMSD to the previously recorded complexes was >1 Å and the interaction energy was within the 5000 most favorable energy complexes recorded. A complex with RMSD <1 Å to a previously recorded complex but lower energy was recorded as a substitute of that complex; higher energy complexes were added to the count of occurrence of the closest recorded complex with lower energy. Finally, we clustered the top 5000 lowest energy structures into 10 groups, which were ranked according to cluster size, taking the number of counts for each complex recorded into account (for details see (54)).

Analysis of docked encounter complexes

The configuration of the LH on the nucleosome was classified for the representative structures of the first two largest clusters of encounter complexes with the highest populations obtained in each docking simulation by applying the following procedure. The nucleosome dyad axis was aligned perpendicular to the viewing plane and the DNA grooves were labeled. The minor groove on the dyad was labeled as 0, and the neighboring major grooves of N-DNA toward L-DNA1 and L-DNA2 were labeled as −1 and +1, respectively. The adjacent major grooves on the L-DNA1 and L-DNA2 were labeled as −2 and +2, respectively, and so on to the ends of the L-DNA arms. The DNA groove contacts of the structural elements of the LH (α_3, β_1, and l_1) were computed for the representative structure of each docking cluster and represented by a vector (see Fig. 3 *A*). The

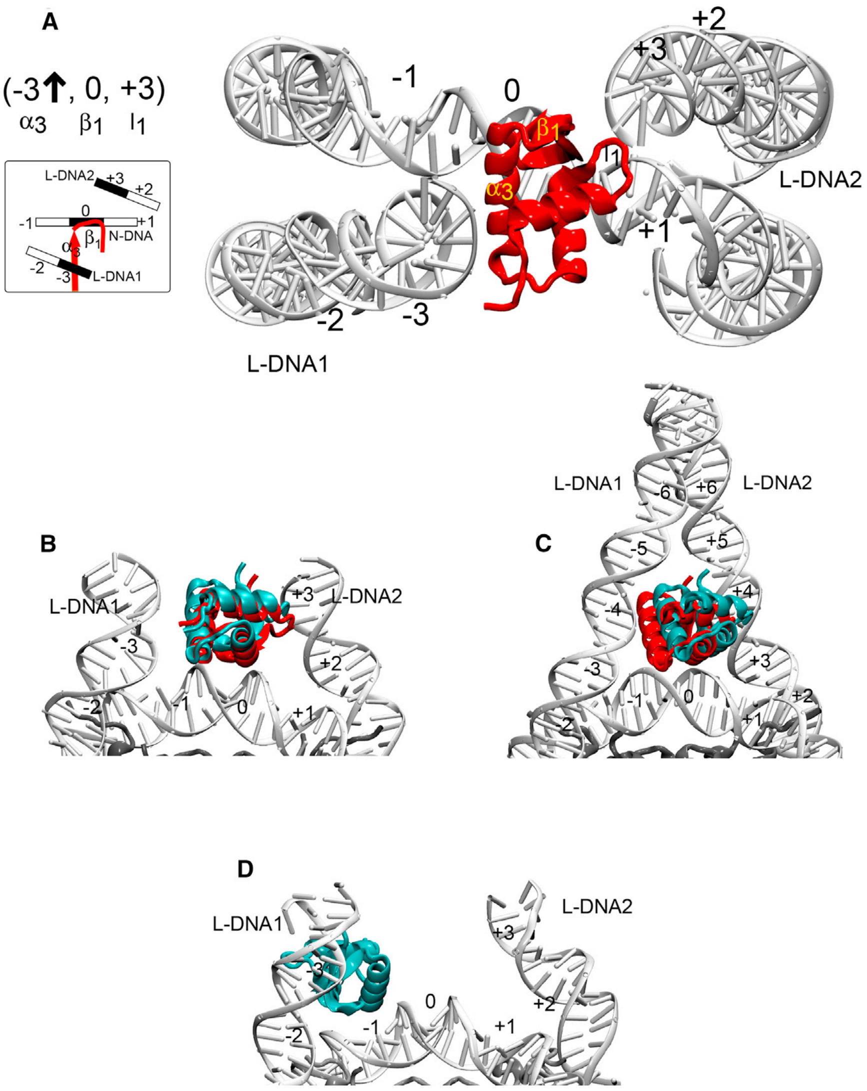

FIGURE 3 Representative LH-nucleosome encounter complexes from BD docking simulations. LHs are shown in cartoon representation and shown in red for reference crystal structures and cyan for docking results. (*A*) The crystal structure of the complex formed by *G. gallus* gH5 bound to a 147 bp Widom 601 DNA sequence nucleosome (PDB: 4QLC) (7) is shown. The classification of the configuration as (−3↑, 0, +3) (on-dyad) is illustrated. (*B*) A representative structure from the largest diffusional encounter-complex cluster (cluster 1) from the docking of *G. gallus* gH5 (residues 24–98) to the mode 7_1 structure of the nucleosome derived by NMA from the crystal structure PDB: 4QLC (7) shown in (*A*). Compared to the position in the crystal structure (*red*), the gH5 has a Cα RMSD of 3.6 Å and the same docked on-dyad configuration (−3↑, 0, +3). (*C*) A representative structure from encounter-complex cluster 2 for *X. laevis* gH1 docked to the nucleosome structure from PDB: 5NL0 (8) is shown. Compared to the position in the crystal structure (*red*), the gH1 has a Cα RMSD of 5.5 Å and the same docked on-dyad configuration (−3↑, 0, +3). (*D*) A representative structure from the encounter-complex cluster with the greatest population (cluster 1) from docking WT *G. gallus* gH5 to snapshot 5 from MD simulation of the nucleosome (see Table 1; Table S6), which represents the average structure in the simulation. The docked configuration is (−1 ↘, −1 −2, −2) and off-dyad. To see this figure in color, go online.

orientation of the α_3-helix of LH was determined, and an arrow was added to the vector to represent the direction of the vector from the N- to the C-terminus of the α_3-helix. An X sign was used when the α_3-helix vector was perpendicular to the viewing plane. See Fig. 3 *A* for an example of the analysis for the crystal structure PDB: 4QLC in the configuration ($-3\uparrow$, 0, $+3$). The PyMOL software (62) was used to quantify hydrogen bonding (with a distance criterion of 3.2–3.6 Å) between the LH and the nucleosome structure.

RESULTS AND DISCUSSION

BD simulations recapitulate experimentally determined LH-nucleosome complex configurations

First, we tested the ability of our protocol of structural refinement of the LH followed by BD rigid-body docking to reproduce the experimentally determined LH-nucleosome structures. In this comparison, it should be borne in mind that the docking protocols generate diffusional encounter complexes that are expected to be close to but not identical to the bound structures studied experimentally. In particular, the rigid-body docked complexes are expected to be looser and will lack optimization of short-range hydrogen bonds and hydrophobic contacts. Therefore, we compared the structures using a classification of the binding configurations based on LH-nucleosome contacts rather than commonly used measures based on RMSD.

Zhou et al. (2015) published the crystal structure of *G. gallus* gH5 bound to a nucleosome with a Widom 601 sequence (PDB: 4QLC) (7). This crystal structure shows an on-dyad binding mode of the gH5. In the current docking simulations and in our previous BD docking study (54), WT *G. gallus* gH5 binds to the nucleosome from the 4QLC structure in an on-dyad configuration. The orientation of gH5 corresponds to that in the crystal structure in the largest encounter-complex cluster (cluster 1) obtained by docking gH5 to the nucleosome of the chromatosome crystal structure PDB: 4QLC (mode 7_0) and to the slightly more open mode 7_1 structure (see Figs. 3 *B* and 4 *A*; Table S3). It should be noted that for the same system in our previous docking simulations (54), we did not apply an LH refinement protocol, and some opening of the nucleosome, as represented by the mode 7_1 and mode 7_2 snapshots, was necessary to allow access of the LH to the nucleosome dyad axis and to reproduce the crystallographic binding mode. This opening of the nucleosome was not necessary for the refined LH structure to bind in the crystallographic binding mode, although binding in this orientation was facilitated by the slight opening in the mode 7_1 structure.

Bednar et al. published the crystal structure of *Xenopus laevis* gH1 bound to a palindromic Widom 601L nucleosome (PDB: 5NL0) (8). This crystal structure also shows

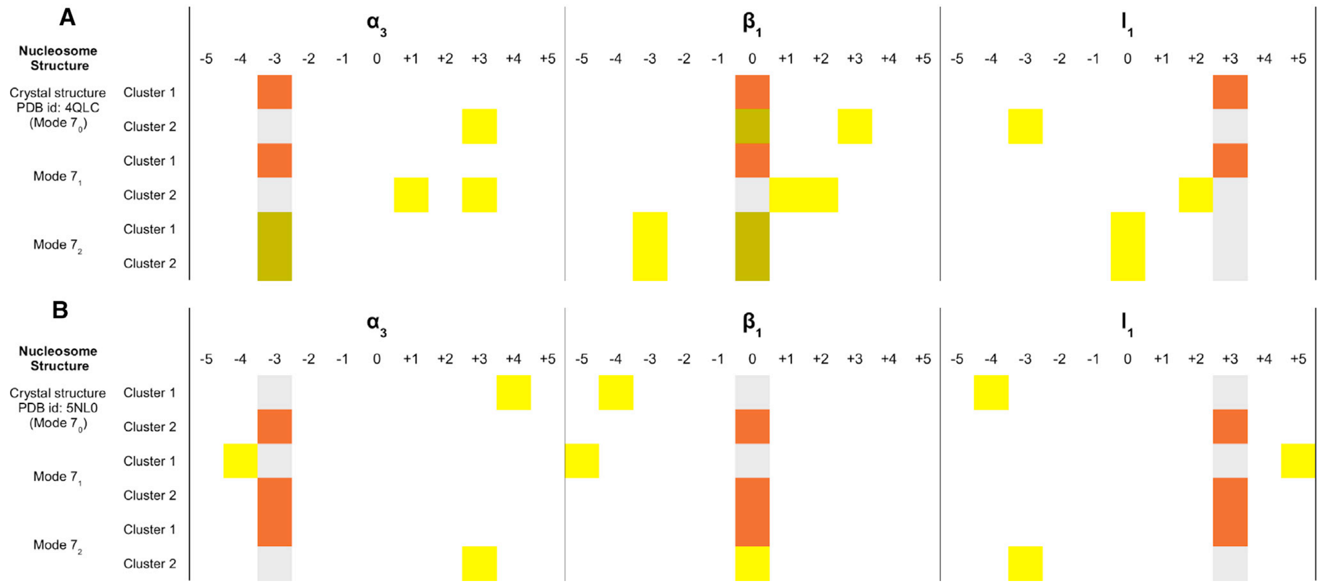

FIGURE 4 Comparison of BD-docked LH GD-nucleosome encounter complexes with crystal structures of the complexes. (*A*) The docking of WT *G. gallus* gH5 to the nucleosome (PDB: 4QLC, Zhou et al. (7)) is shown. (*B*) The docking of WT *X. laevis* gH1 to the nucleosome (PDB: 5NL0, Bednar et al. (8)) is shown. For each system, the orientations of the representative structures of the largest two clusters of docked encounter complexes are given for docking of the LH GD to the nucleosome crystal structure (mode 7_0) and two structures generated by normal mode analysis with slightly opened L-DNA arms (modes 7_1 and 7_2) (see Materials and Methods and Table S2 for details). The DNA grooves on the nucleosome in contact with each structural element of the LH (α_3, β_1, and l_1) are given in the respective columns (see Fig. 3). Color code: the DNA groove contacts of the α_3, β_1, and l_1 elements are given in gray for the crystal structures, in orange when BD results match with the DNA groove contacts of the crystal structures for all three structural elements, in green when the BD results partially (only for one or two structural elements) match with the crystal structures, and in yellow when the BD results have different DNA contacts from the crystal structures. For encounter-complex cluster populations, see Table S3. To see this figure in color, go online.

an on-dyad mode for gH1. Application of our LH refinement and docking protocol reproduced the configuration observed in the crystal structure (-3 ↑, 0, $+3$) in the first- or second-largest encounter-complex cluster when using any of the three nucleosome conformations (modes 7_0–7_2) (Figs. 3 *C* and 4 *B*). Interestingly, the number of encounter complexes observed in each docking simulation was somewhat lower than for the gH5 simulations (1–1.5 million compared to 1.4–2.0 million; see Table S3), indicating that the LH binding site was less accessible, possibly because of the longer L-DNA arms (26 vs. 10 bp). Consistently, in the docking simulations of *X. laevis* gH1 to the crystal structure (mode 7_0) and mode 7_1 of the nucleosome, the α_3-helix of the LH binds to the L-DNA grooves $+4$ and -4, respectively (see Table S3). When the L-DNA arms open further in the mode 7_2 nucleosome structure, the LH can approach closer to the LH core, and the LH α_3-helix binds predominantly to L-DNA groove -3, as observed in the crystal structure. This indicates that further conformational relaxation of the LH and nucleosome should stabilize these on-dyad binding modes.

Summarizing, the diffusional encounter-complex structures generated by BD docking simulations are largely consistent with the crystallographic results of Zhou et al. (7) and Bednar et al. (7,8) for two different LH-nucleosome systems (Fig. 4). We also previously obtained both on- and off-dyad LH binding modes consistent with the available experimental data by BD docking simulations using nucleosome structures generated by normal mode analysis and by MD simulation (53,54). We therefore applied the BD docking approach to investigate the effects of mutations and posttranslational modifications on LH-nucleosome binding configurations.

Single-point mutations in the LH globular domain can significantly affect chromatosome structure

BD docking results for *G. gallus* gH5 and *D. melanogaster* gH1 binding to the eight representative nucleosome structures generated by MD simulation (Figs. S2 and S3) are given in Fig. 5. The nucleosome structures open and close the L-DNA arms to different extents, which were sampled by MD simulation. With respect to the apo-nucleosome structure (snapshot 5), snapshots 1, 2, 3, and 4 are more closed structures, and snapshots 6, 7, and 8 are more open structures (see Figs. S2 and S3 (54)). The binding mode of the largest encounter-complex cluster obtained for the LH variants is compared with that for the WT LH GD for each of the nucleosome structures (see Tables S6 and S7 for the results for the two largest encounter-complex clusters and their populations). Mutant LH-nucleosome configurations that differ significantly from the configurations of the WT LH GD are highlighted in yellow, whereas those that are conserved are highlighted in orange in Fig. 5; gray indicates the configuration obtained from docking the WT LH GD, and green indicates a partial configuration similarity (only for one or two structural elements) with the WT LH.

As found before (53,54), all docked configurations of WT gH5 (with a closed loop) to eight nucleosome

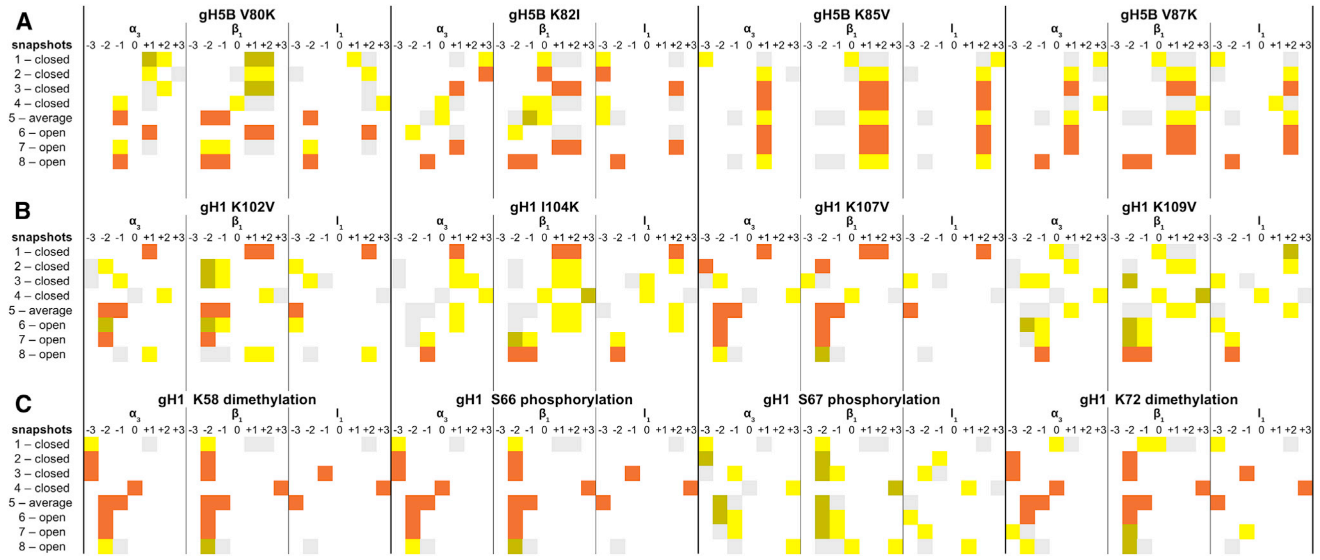

FIGURE 5 Comparison of the BD-docked configurations of *G. gallus* gH5 and *D. melanogaster* gH1 variants to those for WT LH GDs upon docking to eight representative nucleosome structures. (*A*) WT and mutant *G. gallus* gH5, (*B*) WT and mutant *D. melanogaster* gH1, (*C*) and WT and posttranslationally modified *D. melanogaster* gH1 are all shown. The figures show the orientations of the representative structures of the largest cluster of encounter complexes from docking each LH GD to eight representative structures of the nucleosome from an MD simulation started from PDB: 1KX5 (57) (Table 1). The coloring scheme is the same as in Fig. 4. For cluster populations, see Tables S6–S8. To see this figure in color, go online.

structures are off-dyad and in the (−1↘, −1 −2, −2) configuration (Fig. 3 *D*) for the nucleosome structure close to the crystal structure, snapshot 5, and the most open structure, snapshot 8 (Figs. 3 *D* and 5 *A*). The off-dyad LH binding mode is similar to that identified from NMR (9,10) and cryo-electron microscopy (71) experiments. For the other snapshots, the alternative off-dyad docking position is similar to L-DNA1 binding configuration (−1↘, −1 −2, −2), but binding to L-DNA2 (+1↖, +1 +2, +2) dominates, as observed previously (54).

For the gH5 mutants, both off-dyad and on-dyad configurations are observed when considering all eight nucleosome snapshots (Fig. 5 *A*). Interestingly, the on-dyad binding of WT and mutant *G. gallus* gH5 is not observed in the more open nucleosome conformations, suggesting that L-DNA opening is important for the distinction between off- and on-dyad binding modes of the LH. Additionally, the *G. gallus* gH5 K82I mutation resulted in four on-dyad binding modes out of a total of eight docking simulations (see β_1-loop contacts of K82I with DNA groove 0 in Fig. 5 *A*). Considering that the gH5 K82 residue is located at the beginning of the β_1-loop of the LH (Fig. 2 *A*), the unit charge reduction resulting from the isoleucine substitution could reduce contact with the L-DNA arms, making the on-dyad configuration more preferable for this mutant. Moreover, hardly any β_1-loop binding to L-DNA1 is observed for WT and mutant gH5 docking to the more closed nucleosome snapshots. For the most open conformers (snapshots 7 and 8), mainly the off-dyad mode is observed because of the opening of the L-DNA1 arm. Remarkably, mutation of gH5 K85 (which is conserved as lysine at the corresponding position in *D. melanogaster* gH1 and *X. laevis* H1) to valine revealed an off-dyad L-DNA2 binding mode (+1↖, +1 +2, +2) in seven out of eight docking simulations, indicating that, in addition to L-DNA opening angles, LH sequence is also a determinant of the binding configuration.

WT *D. melanogaster* gH1 overall adopts a greater diversity of bound configurations than WT *G. gallus* gH5, but all are off-dyad except for the on-dyad docking mode observed for the most closed nucleosome structure, snapshot 4, and for the docking of the K109V mutant to a closed nucleosome structure, snapshot 1 (Fig. 5 *B*). Interestingly, all gH1 mutants, except K102V, bind on-dyad to snapshot 4. Furthermore, compared to the WT and mutant gH5 simulations, more of the docking poses for WT and mutant gH1 display off-dyad binding to the L-DNA1 arm (Fig. 5, *A* and *B*). These results suggest that *G. gallus* gH5 and *D. melanogaster* gH1 have distinct nucleosome binding preferences. For most snapshots, the number of encounter complexes recorded is lower for gH1 than gH5 (see Tables S6 and S7), indicating lower accessibility to the nucleosome, which also correlates with the higher accessible surface area of gH1 compared to gH5 (3998 vs. 3810 $Å^2$). For the most open structure, snapshot 8, both gH1 and gH5 bind predominantly in the same off-dyad (−1 ↘, −1 −2, −2) configuration (Fig. 5, *A* and *B*).

The effect of LH mutations on the LH GD-nucleosome complex configuration varies among the different snapshots of the nucleosome. For the *G. gallus* gH5 mutants, docking to nucleosome snapshots 1, 2, 4, and 5 resulted in a major configuration shift compared to *G. gallus* gH5 WT for all the mutants (more yellow and less orange in the rows in Fig. 5 *A*). For the *D. melanogaster* gH1 mutants, the LH configuration was most affected (with shifts for all four mutants, more yellow and less orange in the rows in Fig. 5 *B*) compared to WT *D. melanogaster* gH1 for nucleosome snapshot 4, the snapshot with the most closed conformation of the nucleosome. On the other hand, for some snapshots, there were very few shifts in LH-nucleosome configuration upon mutation. For *G. gallus* gH5, only one mutant showed a shift for nucleosome snapshots 6, 7, and 8 (Fig. 5 *A*), whereas for *D. melanogaster* gH1, only one mutant showed a shift in nucleosome snapshot 1 (Fig. 5 *B*). The results show that point mutations may result in a range of changes to LH-nucleosome binding configuration that are dependent on L-DNA opening. The results for the gH5 mutants indicate that chromatosome formation for the more open nucleosome structures may be less sensitive to gH5 sequence, which would have implications for LH binding mechanisms in chromatin, the formation of chromatin structure, and the phenotypic effects of mutations on LHs.

The applied point mutations involved either the introduction or the removal of a +1 charge from the total +11e charge of the two LHs by the exchange of a lysine residue with a hydrophobic residue. Each single-point mutation had a significant effect on LH docking to at least one of the eight different nucleosome structures. This observation is consistent with the idea that LH-nucleosome recognition is strongly affected by electrostatic interaction forces. For *G. gallus* gH5, the total number of configuration changes (number of rows with contacts shown in yellow and green indicating, respectively, no or partial overlap with the WT LH configuration) in the first encounter-complex clusters for docking to the eight different nucleosomes are 5 (V80K) and 4 (K85V, K82I, and V87K), whereas for *D. melanogaster* gH1, they are 7 (K109V), 6 (I104K), 5 (K102V), and 5 (K107V). Previously, by using BD and MD simulations, we showed that *G. gallus* gH5 V87 makes hydrophobic contacts with nucleosome thymine methyl groups in the off-dyad binding mode that are enhanced by induced fit and the adoption of a loop-out conformation of the gH5 (54). Although the rigid-body docking results presented here indicate that the V80, K82, K85, and V87 residues of the *G. gallus* gH5 and the corresponding K102, I104, K107, and K109 residues of *D. melanogaster* gH1 are important for nucleosome recognition, we anticipate that the mutations will also affect stabilization of the chromatosome complex by induced fit.

Analysis of hydrogen bonds between the LH and the phosphate backbone of the DNA indicates that WT and mutant *D. melanogaster* gH1 generally make fewer hydrogen bonds in the encounter complexes compared to WT and mutant *G. gallus* gH5. Summing up the eight different docking simulations and the two largest encounter-complex clusters (Tables S6–S8), WT *D. melanogaster* gH1 makes 27 hydrogen bonds, whereas *G. gallus* gH5 makes 35 hydrogen bonds (Fig. 6; Tables S4 and S5). Interestingly, in WT *D. melanogaster* gH1, the residues making the most hydrogen bonds are K92 and K95 on the α_3-helix, which can bind to alternative DNA grooves on the nucleosome (Fig. 6; Table S5). On the other hand, in *G. gallus* gH5, most of the hydrogen bonds formed in docking simulations are made by R47 and R94 on the α_2-helix and β-sheet, respectively (Fig. 6; Table S4). These hydrogen-bonding differences indicate that different LH isoforms may have different nucleosome recognition mechanisms.

The introduction of single-point mutations in the LHs also resulted in significant changes in hydrogen bonding with the nucleosome. Summing up the eight nucleosome structures of the two largest encounter-complex clusters (Tables S6 and S7), the residues that make more than six hydrogen bonds with the nucleosome in *G. gallus* gH5 mutants are R47 (9 hydrogen bonds (H-bonds) made by V80K mutant and 10 H-bonds made by K85V mutant), K52 (6 H-bonds made by V80K mutant), and K69 (8 H-bonds made by K82I mutant and 12 H-bonds made by V87K mutant) (Fig. 6; Table S4). For *D. melanogaster* gH1 docking, the corresponding residues are K72 (6 H-bonds made by K107V mutant and 7 H-bonds made by K109V mutant) and K91 (6 H-bonds made by K102V mutant) (Fig. 6; Table S5). These results indicate that the hydrogen-bonding network of LH-nucleosome interaction is sensitive to point mutations. Remarkably, the I104K mutant of *D. melanogaster* gH1 makes far fewer H-bonds (18 in eight simulations) compared to the other mutants and PTMs (Fig. 6; Table S5). Interestingly, a significant shift in configuration for *D. melanogaster* gH1 (six of eight encounter complexes shifted compared to WT) is also observed for this mutant, suggests that H-bonding is important for the LH-nucleosome configuration.

In certain LH mutant and nucleosome combinations, single-point mutations on the LH are able to switch the LH binding mode from *D. melanogaster* gH1 to WT *G. gallus* gH5 or vice versa. For example, in docking the I104K mutant of *D. melanogaster* gH1 to nucleosome snapshots 1 and 6, the representative structures from the largest encounter complexes are similar to the configurations for WT *G. gallus* gH5 docking to the same nucleosome conformations (+1 ↖, +1 +2, +2) (Fig. 5, *A* and *B*). Similarly, docking the K109V mutant of the *D. melanogaster* gH1 to

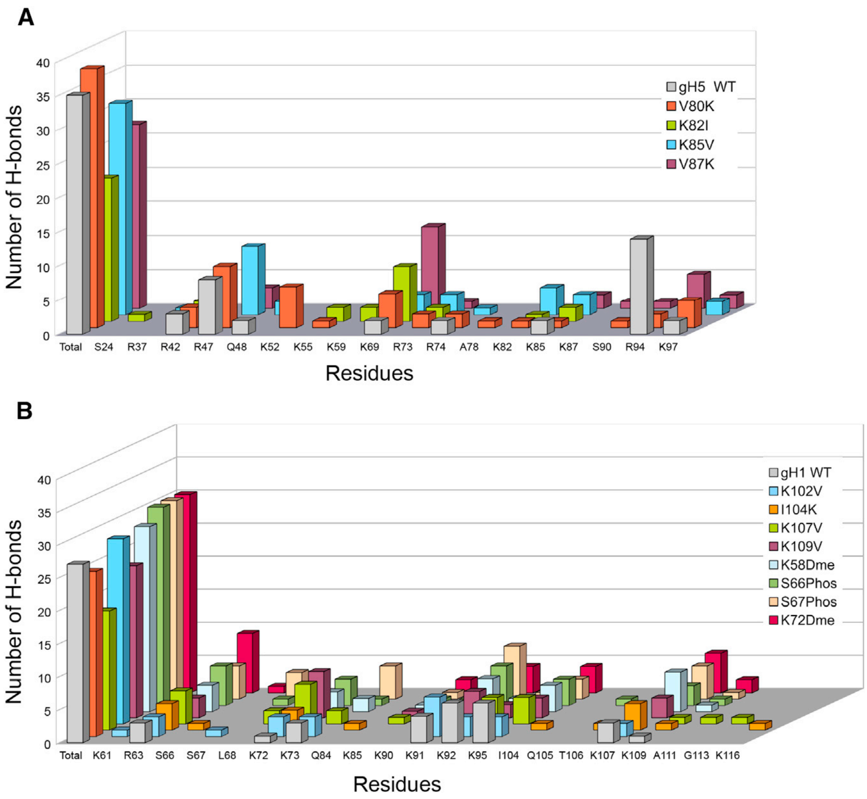

FIGURE 6 The number of H-bonds formed between nucleosomal DNA and LH GDs. The number of H-bonds formed between nucleosomal DNA and (*A*) WT or mutant *G. gallus* gH5 or (*B*) WT, mutant, or posttranslationally modified *D. melanogaster* gH1 are given, summed up over the eight different LH GD-nucleosome docking simulations (each with a different nucleosome conformation) for each LH variant. Some residues make more than one H-bond in the docked position. To see this figure in color, go online.

nucleosome snapshot 6 yielded similar configurations to WT *G. gallus* gH5 for docking to the same nucleosome conformations (+1 ↖, +1 +2, +2) (second encounter complex; Tables S6 and S7). In addition, docking of the K82I mutant of the *G. gallus* gH5 to nucleosome snapshot 6 (cluster 1: (−2 ←, −2, no)) resulted in similar configurations to WT *D. melanogaster* gH1 (Fig. 5, *A* and *B*; Tables S6 and S7). These results indicate that single-point mutations may switch the chromatosome configurations of different LH isoforms.

Even though the sequences of *D. melanogaster* gH1 and *G. gallus* gH5 share 49% sequence identity (Fig. 1), both WT LH GDs docked in the same off-dyad mode to the most open nucleosome conformation, snapshot 8, as (−1 ↘, −1 −2, −2) (Fig. 5, *A* and *B*; Tables S6 and S7). This shows that, apart from the amino-acid sequence of the LH, L-DNA opening of the nucleosome affects the chromatosome configuration. It also suggests that open nucleosome conformations may be able to bind LH proteins off-dyad nonspecifically, and that subsequently more specific on- and off-dyad configurations are formed upon LH-induced nucleosome closing.

PTMs of *D. melanogaster* gH1 can modulate LH-nucleosome binding

In addition to single-point mutations, our docking results revealed that PTMs can also switch the configuration of *D. melanogaster* gH1-nucleosome binding. Four known PTMs, two lysine dimethylations and two serine phosphorylations, were investigated. Dimethylation interferes with salt-link formation, and phosphorylation introduces negative charge. The number of significant shifts in chromatosome configurations (Fig. 5 *C*) upon introducing the PTMs is 8 (S67phosphorylation), 3 (K72dimethylation), and 2 (S66phosphorylation, K58dimethylation). In WT *D. melanogaster* gH1, K58 is on the α_1-helix (Fig. 2 *C*) and has very limited contacts with the DNA in the docked encounter complexes. Thus, it is not surprising that dimethylation of K58 has only a modest effect on nucleosome binding. S66 points toward the LH core, which could explain the limited shifts in configuration observed upon S66phosphorylation (Fig. 2 *C*). On the other hand, S67 and K72 are both on the α_2-helix (Fig. 2 *C*), and introduction of these PTMs at the interaction surface of the LH GD affects the LH binding pose and the number of H-bonds made by the neighboring residues (Fig. 6; Table S5).

Apart from S67phosphorylation, all PTMs resulted in an on-dyad binding mode to the most closed nucleosome conformation, snapshot 4, as observed for WT *D. melanogaster* gH1. Overall, though, for all 40 top-ranked docking encounter complexes for WT gH1 and gH1 with PTMs, 31 resulted in off-dyad binding to L-DNA and only 3 in off-dyad binding to L-DNA2.

For all four PTMs, the number and nature of the H-bonds with the nucleosome compared to WT *D. melanogaster* gH1 is affected for the two most populated encounter complexes (Table S8). For WT *D. melanogaster* gH1, the majority of the H-bonds with the nucleosome are made by K92 and K95 (6 H-bonds each) (Fig. 6; Table S5). For gH1 with PTMs, the most H-bonds are made by R63 (9 H-bonds, K72dimethylation, 6 H-bonds, S66phosphorylation), K91 (8 H-bonds, S67phosphorylation, 6 H-bonds, S66phosphorylation), and K107 (6 H-bonds, K72dimethylation) (Fig. 6; Table S5). Unlike the single-point mutants studied, the gH1 variants with PTMs bind differently to the most open nucleosome conformation, snapshot 8. This may be due to their greater size, which reduces steric accessibility to the N-DNA and results in encounter complexes further from the dyad axis. The high variation in hydrogen bonding upon introducing PTMs suggests that each posttranslationally modified LH could have unique nucleosome interaction features and thus may have a distinct regulatory effect on chromatin compaction and gene regulation.

CONCLUSIONS

By BD docking of refined structures of LH GDs to nucleosome structures, we recapitulated the crystal structures of the complexes determined by Zhou et al. (7) and Bednar et al. (8). These results confirm that BD rigid-body docking is a valid tool for studying LH-nucleosome binding configurations and can be used without prior knowledge of the structural constraints on the structure of the complex. Our previous MD simulations suggested that both conformational selection and induced fit facilitate formation of the bound LH-nucleosome complex (54). Thus, it should be borne in mind that a complete understanding of chromatosome complexation by LH mutants will require further MD simulations to investigate the stability of the fully bound mutant complexes formed from the diffusional encounter complexes generated by BD docking.

The results of our BD docking simulations indicate that the chromatosome configuration is sensitive to single-point mutations and PTMs in the GD of LHs. We found that mutations changing the charge on *G. gallus* gH5 residues V80, K82, K85, and V87 and on *D. melanogaster* gH1 residues K102, I104, K107, and K109 around the LH β-turn significantly affect the LH configuration. The results show that both electrostatic and steric effects of the mutations and PTMs significantly influence the LH-nucleosome configuration. The computed LH GD-nucleosome interaction energies in the diffusional encounter complexes vary within a few kT in the different configurations. Thus, other mutations and PTMs on the nucleosome-binding faces of the LH GDs can be expected to affect LH-nucleosome configuration to varying extents.

Considering the diversity of species of the LHs used in recent experimental studies of LH-nucleosome complexes, our results indicate that a systematic comparison of chromatosome configurations for different LH and nucleosome sequences and single-point mutations is necessary to understand the distribution of the chromatosome structural ensemble and its effect on function. Moreover, experiments to investigate the structural ensemble in solution, such as hydroxyl radical footprinting or NMR, are important to complement crystallographic data. In higher eukaryotes, having a chromatosome structural ensemble could facilitate the ability of one LH isoform to substitute for other LH isoforms, for example, as indicated by recent experimental studies showing that a single LH isoform knockout is not lethal in mice (72).

Currently, there is a significant interest in determining the phenotypic effects of core histone tail PTMs. Here, we show that LH PTMs may alter the chromatosome structural ensemble, which may impact higher-order chromatin structure and possibly gene expression profiles. We found that S67phosphorylation and K72dimethylation cause the most significant shifts in chromatosome configuration, whereas S66phosphorylation and K58dimethylation have modest effects. Applying single-point mutations like K72R to prevent dimethylation (73) and S67E to partially mimic phosphorylation (74) of *D. melanogaster* gH1 could be a promising experimental approach to understand the phenotypic effects of these PTMs.

Our study has certain limitations that need to be borne in mind. First, in our BD simulations, rigid conformations of the molecules are used, and thus possible induced fit mechanisms that could further stabilize the LH-nucleosome complexes are neglected. Furthermore, the behavior of the mutants and PTMs of LHs used in our study could differ from the behavior in vivo because of the presence of the core histone and LH tails as well as the nucleosome connectivity via L-DNA in chromatin. Lastly, the nucleosome structure that we used in MD simulations to investigate off-dyad LH binding could have additional L-DNA conformations relevant to other LH binding modes that were not sampled.

In conclusion, by applying BD docking simulations, we find that the chromatosome structural ensemble is sensitive to specific LH mutations and PTMs, which may have implications for the effects of LH binding on chromatin structure and function.

SUPPORTING MATERIAL

Three figures and eight tables are available at http://www.biophysj.org/biophysj/supplemental/S0006-3495(18)30528-9.

AUTHOR CONTRIBUTIONS

M.A.Ö. carried out all calculations. All authors contributed to the design and analysis of the research and the writing of the manuscript.

ACKNOWLEDGMENTS

We dedicate this work to the memory of Jörg Langowski, who introduced R.C.W. to chromatin biophysics.

M.A.Ö. and R.C.W. thank the Klaus Tschira Foundation and Heidelberg Institute for Theoretical Studies (HITS gGmbH) for funding. V.C. thanks the Max Planck Society, and in particular Hans Schöler, for support.

REFERENCES

1. Olins, A. L., and D. E. Olins. 1974. Spheroid chromatin units (v bodies). *Science.* 183:330–332.
2. Kornberg, R. D. 1974. Chromatin structure: a repeating unit of histones and DNA. *Science.* 184:868–871.
3. Oudet, P., M. Gross-Bellard, and P. Chambon. 1975. Electron microscopic and biochemical evidence that chromatin structure is a repeating unit. *Cell.* 4:281–300.
4. Simpson, R. T. 1978. Structure of the chromatosome, a chromatin particle containing 160 base pairs of DNA and all the histones. *Biochemistry.* 17:5524–5531.
5. Widom, J. 1998. Chromatin structure: linking structure to function with histone H1. *Curr. Biol.* 8:R788–R791.
6. Ramakrishnan, V., J. T. Finch, ..., R. M. Sweet. 1993. Crystal structure of globular domain of histone H5 and its implications for nucleosome binding. *Nature.* 362:219–223.
7. Zhou, B.-R., J. Jiang, ..., Y. Bai. 2015. Structural Mechanisms of Nucleosome Recognition by Linker Histones. *Mol. Cell.* 59:628–638.
8. Bednar, J., I. Garcia-Saez, ..., S. Dimitrov. 2017. Structure and Dynamics of a 197 bp Nucleosome in Complex with Linker Histone H1. *Mol. Cell.* 66:384–397.e8.
9. Zhou, B.-R., H. Feng, ..., Y. Bai. 2013. Structural insights into the histone H1-nucleosome complex. *Proc. Natl. Acad. Sci. USA.* 110:19390–19395.
10. Zhou, B.-R., H. Feng, ..., Y. Bai. 2016. A Small Number of Residues Can Determine if Linker Histones Are Bound On or Off Dyad in the Chromatosome. *J. Mol. Biol.* 428:3948–3959.
11. Orrego, M., I. Ponte, ..., P. Suau. 2007. Differential affinity of mammalian histone H1 somatic subtypes for DNA and chromatin. *BMC Biol.* 5:22.
12. Clausell, J., N. Happel, ..., M. Beato. 2009. Histone H1 subtypes differentially modulate chromatin condensation without preventing ATP-dependent remodeling by SWI/SNF or NURF. *PLoS One.* 4:e0007243.
13. Flanagan, T. W., J. K. Files, K. R. Casano, E. M. George, and D. T. Brown. 2016. Photobleaching studies reveal that a single amino acid polymorphism is responsible for the differential binding affinities of linker histone subtypes H1.1 and H1.5. *Biol. Open.* 5:372–380.
14. George, E. M., T. Izard, ..., D. T. Brown. 2010. Nucleosome interaction surface of linker histone H1c is distinct from that of H1(0). *J. Biol. Chem.* 285:20891–20896.
15. Garg, M., L. R. Perumalsamy, G. V. Shivashankar, and A. Sarin. 2014. The linker histone h1.2 is an intermediate in the apoptotic response to cytokine deprivation in T-effectors. *Int. J. Cell Biol.* 2014:674753.
16. Terme, J.-M., B. Sesé, ..., A. Jordan. 2011. Histone H1 variants are differentially expressed and incorporated into chromatin during differentiation and reprogramming to pluripotency. *J. Biol. Chem.* 286:35347–35357.
17. Happel, N., J. Warneboldt, ..., D. Doenecke. 2009. H1 subtype expression during cell proliferation and growth arrest. *Cell Cycle.* 8:2226–2232.
18. Kowalski, A., and J. Pałyga. 2016. Modulation of chromatin function through linker histone H1 variants. *Biol. Cell.* 108:339–356.
19. Millán-Ariño, L., A. Izquierdo-Bouldstridge, and A. Jordan. 2016. Specificities and genomic distribution of somatic mammalian histone H1 subtypes. *Biochim. Biophys. Acta.* 1859:510–519.

20. Balhorn, R., R. Chalkley, and D. Granner. 1972. Lysine-rich histone phosphorylation. A positive correlation with cell replication. *Biochemistry.* 11:1094–1098.

21. Wiśniewski, J. R., A. Zougman, …, M. Mann. 2007. Mass spectrometric mapping of linker histone H1 variants reveals multiple acetylations, methylations, and phosphorylation as well as differences between cell culture and tissue. *Mol. Cell. Proteomics.* 6:72–87.

22. Garcia, B. A., S. A. Busby, …, D. F. Hunt. 2004. Characterization of phosphorylation sites on histone H1 isoforms by tandem mass spectrometry. *J. Proteome Res.* 3:1219–1227.

23. Lu, A., A. Zougman, …, J. R. Wiśniewski. 2009. Mapping of lysine monomethylation of linker histones in human breast and its cancer. *J. Proteome Res.* 8:4207–4215.

24. Deterding, L. J., M. K. Bunger, …, T. K. Archer. 2008. Global changes in and characterization of specific sites of phosphorylation in mouse and human histone H1 Isoforms upon CDK inhibitor treatment using mass spectrometry. *J. Proteome Res.* 7:2368–2379.

25. Villar-Garea, A., and A. Imhof. 2008. Fine mapping of posttranslational modifications of the linker histone H1 from Drosophila melanogaster. *PLoS One.* 3:e1553.

26. Bonet-Costa, C., M. Vilaseca, …, F. Azorín. 2012. Combined bottom-up and top-down mass spectrometry analyses of the pattern of post-translational modifications of Drosophila melanogaster linker histone H1. *J. Proteomics.* 75:4124–4138.

27. Sarg, B., R. Lopez, …, A. Roque. 2015. Identification of novel post-translational modifications in linker histones from chicken erythrocytes. *J. Proteomics.* 113:162–177.

28. Wiśniewski, J. R., A. Zougman, and M. Mann. 2008. Nepsilon-formylation of lysine is a widespread post-translational modification of nuclear proteins occurring at residues involved in regulation of chromatin function. *Nucleic Acids Res.* 36:570–577.

29. Snijders, A. P. L., S. Pongdam, …, M. J. Dickman. 2008. Characterization of post-translational modifications of the linker histones H1 and H5 from chicken erythrocytes using mass spectrometry. *J. Proteome Res.* 7:4326–4335.

30. Kim, M. Y., T. Zhang, and W. L. Kraus. 2005. Poly(ADP-ribosyl)ation by PARP-1: 'PAR-laying' NAD+ into a nuclear signal. *Genes Dev.* 19:1951–1967.

31. Poirier, G. G., C. Niedergang, …, P. Mandel. 1982. Adenosine diphosphate ribosylation of chicken-erythrocyte histones H1, H5 and high-mobility-group proteins by purified calf-thymus poly(adenosine-diphosphate-ribose) polymerase. *Eur. J. Biochem.* 127:437–442.

32. Jiang, T., X. Zhou, …, P. C. Dedon. 2007. N-formylation of lysine in histone proteins as a secondary modification arising from oxidative DNA damage. *Proc. Natl. Acad. Sci. USA.* 104:60–65.

33. Izzo, A., and R. Schneider. 2016. The role of linker histone H1 modifications in the regulation of gene expression and chromatin dynamics. *Biochim. Biophys. Acta.* 1859:486–495.

34. Horn, P. J., L. M. Carruthers, …, C. L. Peterson. 2002. Phosphorylation of linker histones regulates ATP-dependent chromatin remodeling enzymes. *Nat. Struct. Biol.* 9:263–267.

35. Chubb, J. E., and S. Rea. 2010. Core and linker histone modifications involved in the DNA damage response. *Subcell. Biochem.* 50:17–42.

36. Kim, K., K. W. Jeong, …, W. An. 2012. Functional interplay between p53 acetylation and H1.2 phosphorylation in p53-regulated transcription. *Oncogene.* 31:4290–4301.

37. Happel, N., D. Doenecke, …, T. G. Sourlingas. 2008. H1 histone subtype constitution and phosphorylation state of the ageing cell system of human peripheral blood lymphocytes. *Exp. Gerontol.* 43:184–199.

38. Harshman, S. W., M. E. Hoover, …, M. A. Freitas. 2014. Histone H1 phosphorylation in breast cancer. *J. Proteome Res.* 13:2453–2467.

39. Fischle, W., Y. Wang, and C. D. Allis. 2003. Binary switches and modification cassettes in histone biology and beyond. *Nature.* 425:475–479.

40. Wood, C., A. Snijders, …, M. Dickman. 2009. Post-translational modifications of the linker histone variants and their association with cell mechanisms. *FEBS J.* 276:3685–3697.

41. Collepardo-Guevara, R., G. Portella, …, M. Orozco. 2015. Chromatin Unfolding by Epigenetic Modifications Explained by Dramatic Impairment of Internucleosome Interactions: A Multiscale Computational Study. *J. Am. Chem. Soc.* 137:10205–10215.

42. Grigoryev, S. A., G. Bascom, …, T. Schlick. 2016. Hierarchical looping of zigzag nucleosome chains in metaphase chromosomes. *Proc. Natl. Acad. Sci. USA.* 113:1238–1243.

43. Luque, A., G. Ozer, and T. Schlick. 2016. Correlation among DNA Linker Length, Linker Histone Concentration, and Histone Tails in Chromatin. *Biophys. J.* 110:2309–2319.

44. Luque, A., R. Collepardo-Guevara, …, T. Schlick. 2014. Dynamic condensation of linker histone C-terminal domain regulates chromatin structure. *Nucleic Acids Res.* 42:7553–7560.

45. Perišić, O., and T. Schlick. 2017. Dependence of the Linker Histone and Chromatin Condensation on the Nucleosome Environment. *J. Phys. Chem. B.* 121:7823–7832.

46. Zhou, Y.-B., S. E. Gerchman, …, S. Muyldermans. 1998. Position and orientation of the globular domain of linker histone H5 on the nucleosome. *Nature.* 395:402–405.

47. Bharath, M. M. S., N. R. Chandra, and M. R. S. Rao. 2003. Molecular modeling of the chromatosome particle. *Nucleic Acids Res.* 31:4264–4274.

48. Syed, S. H., D. Goutte-Gattat, …, S. Dimitrov. 2010. Single-base resolution mapping of H1-nucleosome interactions and 3D organization of the nucleosome. *Proc. Natl. Acad. Sci. USA.* 107:9620–9625.

49. van Zundert, G. C. P., J. P. G. L. M. Rodrigues, …, A. M. J. J. Bonvin. 2016. The HADDOCK2.2 Web Server: User-Friendly Integrative Modeling of Biomolecular Complexes. *J. Mol. Biol.* 428:720–725.

50. Trott, O., and A. J. Olson. 2010. AutoDock Vina: improving the speed and accuracy of docking with a new scoring function, efficient optimization, and multithreading. *J. Comput. Chem.* 31:455–461.

51. Gabdoulline, R. R., and R. C. Wade. 1997. Simulation of the diffusional association of barnase and barstar. *Biophys. J.* 72:1917–1929.

52. Gabdoulline, R. R., and R. C. Wade. 1998. Brownian dynamics simulation of protein-protein diffusional encounter. *Methods.* 14:329–341.

53. Pachov, G. V., R. R. Gabdoulline, and R. C. Wade. 2011. On the structure and dynamics of the complex of the nucleosome and the linker histone. *Nucleic Acids Res.* 39:5255–5263.

54. Öztürk, M. A., G. V. Pachov, …, V. Cojocaru. 2016. Conformational selection and dynamic adaptation upon linker histone binding to the nucleosome. *Nucleic Acids Res.* 44:6599–6613.

55. Papamokos, G. V., G. Tziatzos, …, E. Kaxiras. 2012. Structural role of RKS motifs in chromatin interactions: a molecular dynamics study of HP1 bound to a variably modified histone tail. *Biophys. J.* 102:1926–1933.

56. Erler, J., R. Zhang, …, J. Langowski. 2014. The role of histone tails in the nucleosome: a computational study. *Biophys. J.* 107:2911–2922.

57. Hendzel, M. J., M. A. Lever, …, J. P. H. Th'ng. 2004. The C-terminal domain is the primary determinant of histone H1 binding to chromatin in vivo. *J. Biol. Chem.* 279:20028–20034.

58. Lindahl, E., C. Azuara, …, M. Delarue. 2006. NOMAD-Ref: visualization, deformation and refinement of macromolecular structures based on all-atom normal mode analysis. *Nucleic Acids Res.* 34:W52–W526.

59. Heo, L., H. Park, and C. Seok. 2013. GalaxyRefine: Protein structure refinement driven by side-chain repacking. *Nucleic Acids Res.* 41:W384–W388.

60. Davey, C. A., D. F. Sargent, …, T. J. Richmond. 2002. Solvent mediated interactions in the structure of the nucleosome core particle at 1.9 a resolution. *J. Mol. Biol.* 319:1097–1113.

61. Schalch, T., S. Duda, …, T. J. Richmond. 2005. X-ray structure of a tetranucleosome and its implications for the chromatin fibre. *Nature.* 436:138–141.

62. The PyMOL Molecular Graphics System, Version 1.721 Schrödinger, LLC.

63. Warnecke, A., T. Sandalova, …, R. A. Harris. 2014. PyTMs: a useful PyMOL plugin for modeling common post-translational modifications. *BMC Bioinformatics.* 15:370.

64. Chung, H. H., S. K. Sze, …, V. C.-L. Lin. 2014. Lysine methylation of progesterone receptor at activation function 1 regulates both ligand-independent activity and ligand sensitivity of the receptor. *J. Biol. Chem.* 289:5704–5722.

65. Homeyer, N., A. H. C. Horn, …, H. Sticht. 2006. AMBER force-field parameters for phosphorylated amino acids in different protonation states: phosphoserine, phosphothreonine, phosphotyrosine, and phosphohistidine. *J. Mol. Model.* 12:281–289.

66. Dolinsky, T. J., P. Czodrowski, …, N. A. Baker. 2007. PDB2PQR: expanding and upgrading automated preparation of biomolecular structures for molecular simulations. *Nucleic Acids Res.* 35:W522-5.

67. Ponder, J. W., and D. A. Case. 2003. Force fields for protein simulations. *Adv. Protein Chem.* 66:27–85.

68. Holst, M., N. Baker, and F. Wang. 2000. Adaptive multilevel finite element solution of the Poisson–Boltzmann equation I. Algorithms and examples. *J. Comput. Chem.* 21:1319–1342.

69. Gabdoulline, R. R., and R. C. Wade. 1996. Effective Charges for Macromolecules in Solvent. *J. Phys. Chem.* 100:3868–3878.

70. Martinez, M., N. J. Bruce, …, R. C. Wade. 2015. SDA 7: A modular and parallel implementation of the simulation of diffusional association software. *J. Comput. Chem.* 36:1631–1645.

71. Song, F., P. Chen, …, G. Li. 2014. Cryo-EM study of the chromatin fiber reveals a double helix twisted by tetranucleosomal units. *Science.* 344:376–380.

72. Fan, Y., T. Nikitina, …, A. I. Skoultchi. 2003. H1 linker histones are essential for mouse development and affect nucleosome spacing in vivo. *Mol. Cell. Biol.* 23:4559–4572.

73. Jin, Y., A. M. Rodriguez, …, J. J. Wyrick. 2007. Simultaneous mutation of methylated lysine residues in histone H3 causes enhanced gene silencing, cell cycle defects, and cell lethality in Saccharomyces cerevisiae. *Mol. Cell. Biol.* 27:6832–6841.

74. Oueslati, A., K. E. Paleologou, …, H. A. Lashuel. 2012. Mimicking phosphorylation at serine 87 inhibits the aggregation of human α-synuclein and protects against its toxicity in a rat model of Parkinson's disease. *J. Neurosci.* 32:1536–1544.

Biophysical Journal, Volume *114*

Supplemental Information

Dependence of Chromatosome Structure on Linker Histone Sequence and Posttranslational Modification

Mehmet Ali Öztürk, Vlad Cojocaru, and Rebecca C. Wade

Supplementary Figure 1: Structure based pairwise sequence alignments of the DNA in the nucleosomes used in this study. The systems studied are listed in Table 1 and referred to by the PDB identifier. The L-DNA1 and L-DNA2 linker DNA arms are labeled by bold and the nucleotides at the dyad point are highlighted in green. Pairwise alignments are shown as 4QLC and 5NL0 are very similar (51% sequence identity) while 1KX5/1ZBB is rather different in sequence (42% sequence identity to 4QLC and 31% to 5NL0).

```
4QLC         L-DNA2 ---------------ACTGGCCGCCCTGGAGAATCCCGGTGCCGAGGCCGCTCAATTGGT    45
5NL0         L-DNA2 ACTACGTAATATTGGCCAGCTAGGATATCACAATCCCGGTGCCGAGGCCGCTCAATTGGT    60
                                    * *   *      * *****************************

4QLC                CGTAGACAGCTCTAGCACCGCTTAAACGCACGTACGCGCTGTCCCCCGCGTTTTAACCGC   105
5NL0                CGTAGACAGCTCTAGCACCGCTTAAACGCACGTACGGAATCCGTACGTGCGTTTAAGCGG   120
                    ************************************   *     *     ***** **

4QLC                CAAGGGGATTACTCCCTAGTCTCCAGGCACGTGTCAGATATATACATCCTGTGCATGTAA   165
5NL0                TGCTAGAGCTGTCTACGACCAATTGAGCGGCCTCGGCACCGGGATTGTGATATCCTAGCT   180
                         *   *     * *        **                           *

4QLC                GT--------------- L-DNA1 167
5NL0                GGCCAATATTACGTAGT L-DNA1 197
                    *


4QLC         L-DNA2 ACTGGCCGCCCTGGAGAATCCCGGTGCCGAGGCCGCTCAATTGGTCGTAGACAGCTCTAG    60
1KX5/1ZBB    L-DNA2 ACTGGCCGCCCTGGAGAATCACCTGCAGATACTACCAAAAGTGTATTTGGAAACTGCTCC    60
                    ******************** *             *  ** **    * ** *   **

4QLC                CACCGCTTAAACGCACGTACGCGCTGTCCCCCGCGTTTTAACCGCCAAGGGGATTACTCC   120
1KX5/1ZBB           ATCAAAAGGCATGTTCAGCTGGAATCCAGCTGAACATGCCTTTTGATGGAGCAGTTTCCA   120
                      *         *  *    *   *    *      *           * * * *   *

4QLC                CTAGTCTCCAGGCACGTGTCAGATATATACATCCTGTGCATGTAAGT L-DNA1         167
1KX5/1ZBB           AATACACTTTTGGTAGTATCTGCAGGTTACATCCTGTGCATGTAAGT L-DNA1         167
                               *   ** ** *     ********************
```

```
5NL0       L-DNA2 ACTACGTAATATTGGCCAGCTAGGATATCACAATCCCGGTGCCGAGGCCGCTCAATTGGT  60
1KX5/1ZBB  L-DNA2 ---------------ACTGGCCGCCCTGGAGAATCACCTGCAGATACTACCAAAAGTGTA  60
                                  * *   *      * **** *             *  ** **

5NL0              CGTAGACAGCTCTAGCACCGCTTAAACGCACGTACGGAATCCGTACGTGCGTTTAAGCGG  120
1KX5/1ZBB         TTTGGAAACTGCTCCATCAAAAGGCATGTTCAGCTGGAATCCAGCTGAACATGCCTTTTG  120
                    * ** *   **    *       * *  *    *******    *  * *       *

5NL0              TGCTAGAGCTGTCTACGACCAATTGAGCGGCCTCGGCACCGGGATTGTGATATCCTAGCT  180
1KX5/1ZBB         ATGGAGCAGTTTCCAAATACACTTTTGGTAGTATCTGCAGGTTACATCCTGTGCATGTAA  167
                      **   * ** *    ** **  *             *  *         * *

5NL0              GGCCAATATTACGTAGT L-DNA1 197
1KX5/1ZBB         GT--------------- L-DNA1 167
                  *
```

Supplementary Figure 2: Calculation of L-DNA opening angles. **A-** The vectors v_L^1 and v_L^2 were defined based on selected DNA bases to represent the helical axes of L-DNA1 and L-DNA2, respectively, Öztürk et al. (3). The double headed arrows show the directionality of the L-DNA motions described by the two angles (γ_1 and γ_2). **B-** 8 Snapshots selected from a 100 ns standard MD simulation of the free nucleosome used for BD simulations (labeled in red) on the γ_1 and γ_2 histograms for L-DNA1 and L-DNA2, Öztürk et al. (3). Snapshots 1-4 have more closed conformations of the nucleosome and snapshots 6-8 have more open conformations of the nucleosome compared to snapshot 5, see Supplementary Figure 3. (Figure S2B is re-printed from Öztürk et al. (3) under Open access CC BY license.)

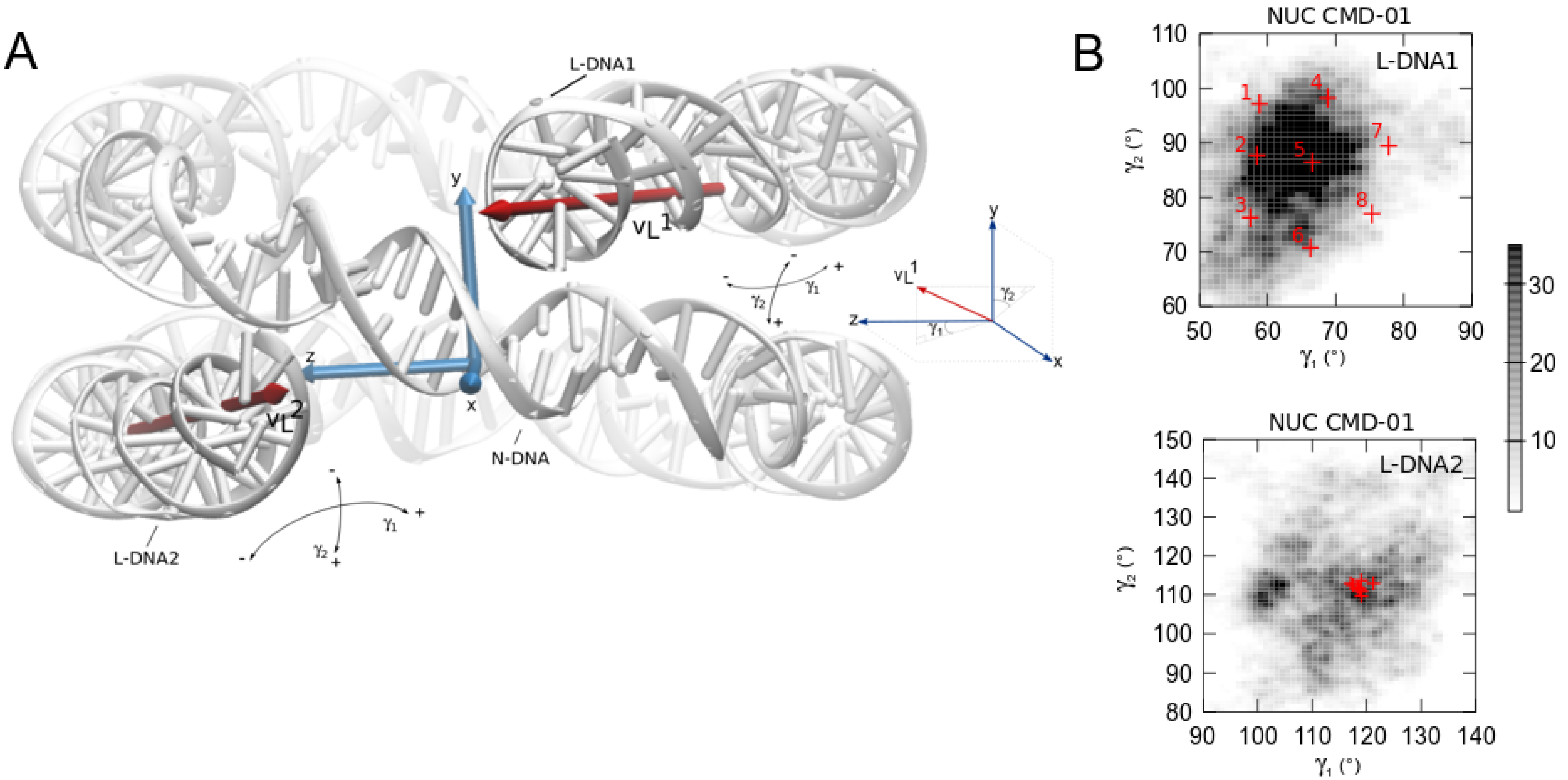

Supplementary Figure 3: Superposition of the 8 nucleosome snapshots selected from 100 ns standard MD simulation of the nucleosome for BD simulations (see Supplementary Figure 2). The DNA is colored according to snapshot and the core histones are shown in cartoon representation in gray. Snapshots 1-4 (1-blue, 2-green, 3-cyan, 4-lime) have more closed conformations of the nucleosome and snapshots 6-8 (6-pale pink, 7-red, 8-magenta) have more open conformations of the nucleosome compared to snapshot 5 (orange).

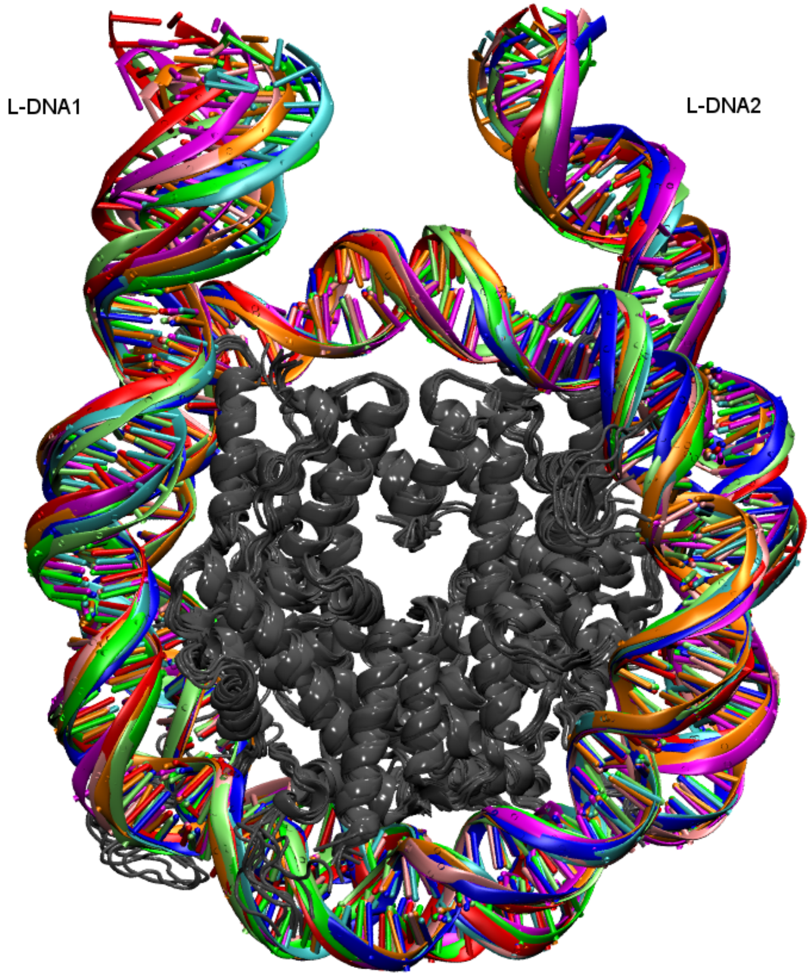

Supplementary Table 1 L-DNA opening angles are given for each L-DNA arm for the nucleosome structures used for the BD docking simulations with results given in Figure 5. The L-DNA1 arm is in a relatively closed form in nucleosome structures 1, 2, 3 and 4 and in a more open form for nucleosome structures 6, 7 and 8 (see Supplementary Figure 2 and reference (3) for further details.).

Nucleosome structure	L-DNA1 γ_1(°)	L-DNA1 γ_2(°)	L-DNA2 γ_1(°)	L-DNA2 γ_2(°)
**1**	58.7	97.0	119.0	109.7
**2**	58.4	87.6	118.3	111.8
**3**	57.4	76.2	118.7	111.5
**4**	68.8	98.2	119.0	110.9
**5**	66.5	86.4	121.2	112.9
**6**	67.2	77.2	118.6	110.0
**7**	77.7	89.4	117.0	112.9
**8**	75.2	76.9	118.9	113.7

The motions of the L-DNAs were described using the angles γ_1 and γ_2, where γ_1 = the angle between the xz projection of the vector v_L^1 or v_L^2 and the z axis, and γ_2 = the angle between the xy projection of v_L^1 or v_L^2 and the y axis. v_L^1 and v_L^2 were defined based on selected DNA residues along the helical axis of the two L-DNAs (See Supplementary Figure 2). v_L^1 connects the geometric centers of nucleotides 12-15, 320-323 and 2-5, 330-333, whereas v_L^2 connects the geometric centers of nucleotides 153-156, 179-182 and 163-166, 169-172. The numbering of the DNA nucleotides starts from 1 and 168 at the 5' ends of L-DNA1 and L-DNA2, respectively, and runs to 167 and 334 at the 3' ends of L-DNA2 and L-DNA1, respectively. All non-hydrogen atoms were used to define the nucleotides.

Supplementary Table 2 L-DNA opening angles are given for each L-DNA arm for the conformations derived from NMA using the nucleosome from the crystal structure with PDB id: 4QLC determined by Zhou et al. (1) and PDB id: 5NL0 determined by Bednar et al. (2). The conformations were generated along the first internal motion mode (mode 7, modes 1-6 correspond to rigid body translation and rotation). Mode 7_0 corresponds to the crystal structure. Mode 7_1 and mode 7_2 represent increasingly more open structures of the nucleosome. These three structures were used in BD docking to obtain the results given in Figure 4. See Supplementary Figure 2 for the definitions of the angles.

**Nucleosome structure**	**L-DNA1 γ_1(°)**	**L-DNA1 γ_2(°)**	**L-DNA2 γ_1(°)**	**L-DNA2 γ_2(°)**
**Crystal structure (4QLC) (Mode 7_0)**	56.0	98.9	111.2	78.3
**Mode 7_1**	57.1	99.8	105.7	77.4
**Mode 7_2**	58.5	101.9	99.9	76.2
**Crystal structure (5NL0) (Mode 7_0)**	60.5	84.5	108.7	83.7
**Mode 7_1**	62.4	85.2	105.6	82.5
**Mode 7_2**	64.1	85.9	102.6	81.3

The vectors for calculating the L-DNA angles were defined as described in Supplementary Table 1 using the corresponding aligned DNA bases.

Supplementary Table 3 BD docking simulations of LH binding to the nucleosome structures derived from the crystal structures with PDB ids: 4QLC (1) and 5NL0 (2). The orientations of the representative structures of the largest two clusters of encounter complexes obtained by docking WT *G. gallus* gH5 to the Zhou et al. (PDBid: 4QLC) nucleosome and WT *X. laevis* gH1 docking to the Bednar et al. nucleosome (PDB id: 5NL0) are given for docking to the nucleosome crystal structure (Mode 7_0) and two structures (Mode 7_1 and Mode 7_2) with slightly opened L-DNA arms.

Nucleosome structure (*)	gH5 WT					Nucleosome structure (*)	gH1 WT				
	N	%	α_3	β_1	l_1		N	%	α_3	β_1	l_1
Reference (4QLC)			-3 ↑	0	+3	Reference (5NL0)			-3 ↑	0	+3
Crystal structure (4QLC) (Mode 7_0)	2.0	35	-3 ↑	0	+3	Crystal structure (5NL0) (Mode 7_0)	1.5	39	+4 ↖	-4	-4
		31	+3 ↘	0, +3	-3			27	-3 ↑	0	+3
Mode 7_1	1.4	79	-3 ↑	0	+3	Mode 7_1	1.2	46	-4 →	-5	+5
		8	+1, +3 ↑	+1, +2	+2			34	-3 ↑	0	+3
Mode 7_2	2.0	36	-3 ↑	0, -3	0	Mode 7_2	1.0	85	-3 ↑	0	+3
		28	-3 ↑	0, -3	0			8	+3 ↓	0	-3

The DNA grooves on the nucleosome in contact with each structural element of LH (α_3, β_1 and l_1) are given in the respective columns (See Figure 3A). The arrows show the orientation of the LH α_3-helix when the nucleosome is aligned perpendicular to the viewing plane. N is the total number of encounter complexes that satisfy the docking criteria in each BD simulation, divided by 10^6. The % of these encounter complexes in the two largest BD clusters is given in the % column for clusters 1 (above) and 2 (below). (*) Normal Mode Analysis of an elastic network model of the nucleosome was performed. The crystal structure corresponds to mode 7_0 and modes 7_1 and 7_2 are structures along the slowest mode (mode 7) that have more open L-DNA arms than the crystal structure (See Methods and Supplementary Table 2 for details.)

Supplementary Table 4 The number of hydrogen bonds formed between nucleosomal DNA and wild-type and mutant *G. gallus* gH5 are summed over the 8 different *G. gallus* gH5 - nucleosome docking simulations, each using a different nucleosome conformation. Occurrences of 6 or more hydrogen bonds to a *G. gallus* gH5 residue are highlighted in red. Some residues have more than one hydrogen bond in the docked position.

Residues	gH5 WT	V80K	K82I	K85V	V87K
**S24**	0	0	1	0	0
**R37**	0	0	0	1	0
**R42**	3	3	3	2	0
**R47**	**8**	**9**	0	**10**	3
**Q48**	2	0	0	2	0
**K52**	0	**6**	0	0	0
**K55**	0	1	2	0	0
**K59**	0	0	2	0	0
**K69**	2	5	**8**	3	**12**
**R73**	0	2	2	3	1
**R74**	2	2	0	1	0
**A78**	0	1	0	0	0
**K82**	0	1	1	4	0
**K85**	2	1	2	3	2
**K87**	0	0	0	0	1
**S90**	0	1	0	0	1
**R94**	**14**	2	0	0	5
**K97**	2	4	0	2	2
**Total**	**35**	**38**	**21**	**31**	**27**

Supplementary Table 5 The number of hydrogen bonds formed between nucleosomal DNA and wild-type, mutant and post-translationally modified *D. melanogaster* gH1 summed over the 8 different *D. melanogaster* gH1 - nucleosome docking simulations, each using a different nucleosome conformation. Occurrences of 6 or more hydrogen bonds to a *D. melanogaster* gH1 residue are highlighted in red. Some residues have more than one hydrogen bond in the docked position.

Residues	gH1 WT	K102V	I104K	K107V	K109V	K58Dme	S66Phos	S67Phos	K72Dme
**K61**	0	1	0	0	0	0	0	0	0
**R63**	3	3	4	5	3	4	**6**	5	**9**
**S66**	0	0	1	0	0	0	0	0	1
**S67**	0	1	0	0	0	0	1	4	0
**L68**	0	0	0	2	0	0	0	0	0
**K72**	1	3	3	**6**	**7**	3	4	0	0
**K73**	3	3	0	2	0	2	1	5	0
**Q84**	0	0	1	0	0	0	0	0	0
**K85**	0	0	0	1	1	1	1	1	2
**K90**	0	0	0	0	0	0	1	0	0
**K91**	4	**6**	0	1	4	5	**6**	**8**	4
**K92**	**6**	3	1	4	2	2	1	1	1
**K95**	**6**	3	0	4	3	4	4	3	4
**I104**	0	0	1	0	0	0	0	0	0
**Q105**	0	0	0	0	0	0	1	0	0
**T106**	0	0	1	0	0	0	0	0	1
**K107**	3	2	4	0	3	**6**	3	5	**6**
**K109**	1	0	1	1	0	1	1	1	2
**A111**	0	0	0	1	0	0	0	0	0
**G113**	0	0	0	1	0	0	0	0	0
**K116**	0	0	1	0	0	0	0	0	0
**Total**	**27**	**25**	**18**	**28**	**23**	**28**	**30**	**30**	**30**

Supplementary Table 6 Docked configurations of WT and mutant *G. gallus* gH5 – nucleosome encounter complexes. The two largest encounter complexes are listed. Docking was performed to the 8 representative structures of the nucleosome from an MD simulation started from PDB id:1KX5. For details, see Table 1.

Snapshot	gH5B WT					gH5B V80K					gH5B K82I					gH5B K85V					gH5B V87K				
	N	%	α_3	β_1	l_1	N	%	α_3	β_1	l_1	N	%	α_3	β_1	l_1	N	%	α_3	β_1	l_1	N	%	α_3	β_1	l_1
1	2.7	68	+1 ↖	+1,+2	+2	3.8	46	+1 +2 X	+1,+2	+1	7.6	51	+3 ↓	0	-3	5.3	46	-3 ↑	0	+3	4.5	94	+3 ↓	0	-3
		29	-3 ↑	0	+2		35	+1 ↖	+1,+2	+2		9	+3 ↓	0	-3		23	0 ↙	0	+2		3	+3 ↓	0	-3
2	2.8	42	+3 ↓	0	-3	4.5	82	+1 ↑	+1,+2	+2	4.2	26	+3 ↓	0	-3	7.3	59	+1 ↖	+1,+2	+2	3.3	62	+1 ↖	+1,+2	+2
		42	+1 ↖	+1,+2	+2		8	+1, +2 ↙	+1,+2	+3		26	+3 ↓	0	-3		32	+1 ↖	+1,+2	+2		28	-2 ↘	-1,-2	-3
3	5.6	53	+1 ↖	+1,+2	+2	5.7	39	+2 X	+1,+2	no	2.1	59	+1 ↖	+1,+2	+2	6.4	38	+1 ↖	+1,+2	+2	5.4	95	+1 ↖	+1,+2	+2
		37	+1 ↖	+1,+2	+2		20	+2 X	+1,+2	no		28	0 ↓	0, -1	-3		25	+1 ↖	+1,+2	+2		2	-2 ↘	-1,-2	-3
4	3.0	67	+1 ↖	+1,+2	+2	2.1	39	-1 ↑	0	+3	4.1	40	0 ↓	0, -1	-3	3.9	48	+1 ↖	+1,+2	+2	3.9	48	+3 ↙	+3	+1
		17	0, +3 ↓	0, -1	no		28	+3 ↓	0	no		17	0 ↓	0, -1	-3		25	0 ↙	+1	+3		40	+1 ↖	+1,+2	+2
5	4.9	38	-1 ↘	-1,-2	-2	3.7	28	-1 ↘	-1,-2	-2	4.8	45	0 ↓	0, -1	-3	13.9	37	+1 ↖	+1,+2	+2	7.5	48	+1 ↖	+1,+2	+2
		24	-1 ↘	-1,-2	-2		21	+1, +2↙	+1,+2	no		14	0 ↓	0	-3		25	+1 ↖	+1,+2	+2		31	0 ↓	0	-3
6	3.3	58	+1 ↖	+1,+2	+2	5.4	61	+1↖	+1,+2	+2	1.6	85	-2 ←	-2	no	4.3	86	+1 ↖	+1,+2	+2	4.1	73	+1 ↖	+1,+2	+2
		29	+1 ↖	+1,+2	+2		16	+1↖	+1,+2	+2		6	+2 ←	+2	no		6	+1,+2 ↖	+1,+2	no		15	0 ↑	0	+2
7	7.4	27	+1 ↖	+1,+2	+2	9.4	41	-1 ↘	-1,-2	-2	0.7	42	+1↖	+1	+2	8.1	26	+1 ↖	+1,+2	+2	3.1	96	+1 ↖	+1,+2	+2
		20	+1 ↖	+1,+2	+2		37	-1 ↘	-1,-2	-2		31	-1 ↓	0	no		24	+1 ↖	+1,+2	+2		2	-1 ↘	-1,-2	-2
8	5.0	42	-1 ↘	-1,-2	-2	5.6	74	-1 ↘	-1,-2	-2	2.6	33	-1 ↘	-1,-2	-2	6.1	44	+1 ↖	+1,+2	+2	5.0	42	-1 ↘	-1,-2	-2
		28	-1 ↘	-1,-2	-2		10	-1 ↙	-1	-2		23	-1 ↘	-1,-2	-2		16	-1 ↘	-1,-2	-2		28	-1 ↘	-1,-2	-2

Supplementary Table 7 Docked configurations of WT and mutant *D. melanogaster* gH1 – nucleosome encounter complexes. The two largest encounter complexes are listed. Docking was performed to 8 representative structures of the nucleosome from an MD simulation started from PDB id:1KX5. For details, see Table 1.

Snapshot	gH1 WT					gH1 K102V					gH1 I104K					gH1 K107V					gH1 K109V				
	N	%	α_3	β_1	l_1	N	%	α_3	β_1	l_1	N	%	α_3	β_1	l_1	N	%	α_3	β_1	l_1	N	%	α_3	β_1	l_1
1	2.3	25	+1↖	+1, +2	+2	1.9	50	+1↖	+1,+2	+2	2.4	66	+1↖	+1,+2	+2	1.1	39	+1↖	+1,+2	+2	1.6	47	0↑	0	+2
		21	-3↖	-3	no		21	0↓	-1,-3	-3		9	+1↖	+1,+2	+2		25	0, -1↓	-1,-2	no		28	0↑	0	+2
2	3.3	97	-3←	-2	no	1.8	49	-2↗	-1,-2	-3	3.9	72	+1↖	+1,+2	+2	1.2	57	-3↙	-2	no	1.8	70	+1↖	+1,+2	+2
		2	-1↙	-1,-2	-3		20	+1↖	+1,+2	+2		15	-2→	-1,-2	-2		34	-2↗	-3	-3		15	-2↗	-1, -2	-3
3	3.9	60	-3←	-2	-1	2.7	27	-1↙	-1,-2	-2	4.4	65	+1,+2↓	+1,+2	0	1.7	55	-2↗	-3	-3	2.2	54	-1, -2↗	-2	-3
		31	-1↙	-1,-2	-2		26	-1, -2↖	-1,-2	0		21	-1↘	-1,-2	-2		22	0, -1↙	-1	-3		23	+2↓	+1,+2	no
4	2.7	83	0↖	+3	+3	1.3	47	+2→	+2	no	3.1	92	+3↙	0,+3	0	0.9	42	+3↙	0	no	0.9	69	+3↙	0,+3	0
		4	-3↖	-2	-1		20	-3↖	-1	+3		6	+1↖	+1,+2	+2		25	-3↗	-1	no		14	-1↗	0	+3
5	4.0	59	-1, -2↗	-1, -2	-3	4.9	36	-1, -2↗	-1,-2	-3	4.5	51	+1↖	+1,+2	+2	3.5	22	-1, -2↗	-1,-2	-3	1.8	53	+1↖	+1,+2	+2
		27	-2↖	-2	no		28	-1, -2↗	-1,-2	-3		35	-1, -2→	-1,-2	-2		21	-1, -2↗	-1,-2	-3		28	-1, -2↗	-1,-2	-3
6	1.9	63	-2←	-2	no	2.1	55	-2↗	-1,-2	-3	5.3	95	+1↖	+1,+2	+2	3.9	55	-2←	-2	no	2.3	32	-1, -2↗	-1,-2	-3
		29	-1,-2↗	-1,-2	-3		13	-1, -2↗	-1,-2	-3		1	-1↘	-1,-2	-2		24	-1, -2↗	-1,-2	-3		32	+1↖	+1,+2	+2
7	1.5	49	-2←	-2	no	1.9	74	-2←	-2	no	5.8	27	-1↘	-1,-2	-2	2.6	44	-2←	-2	no	1.9	78	-1↘	-1,-2	-2
		22	-3↖	-2	-1		14	+1↖	+1,+2	+2		24	-1↘	-1,-2	-2		30	-2←	-2	no		12	-1↘	-1,-2	-2
8	4.0	75	-1↘	-1,-2	-2	2.2	64	+1↖	+1,+2	+2	3.6	61	-1↘	-1,-2	-2	3.3	29	-2←	-2	no	1.8	77	-1↘	-1,-2	-2
		8	-1↘	-1,-2	-2		19	-1↘	-1,-2	-2		28	-1↘	-1,-2	-2		28	-1, -2↗	-1,-2	-3		9	-1,-2↘	-1,-2	-3

Supplementary Table 8 Docking configurations of WT and post-translationally modified *D. melanogaster* gH1 – nucleosome encounter complexes. The two largest encounter complexes are listed. Docking was performed to 8 representative structures of the nucleosome from an MD simulation started from PDB id:1KX5. For details, see Table 1.

Snapshot	gH1 WT					gH1 K58 dimethylation					gH1 S66 phosphorylation					gH1 S67 phosphorylation					gH1 K72 dimethylation				
	N	%	α_3	β_1	l_1	N	%	α_3	β_1	l_1	N	%	α_3	β_1	l_1	N	%	α_3	β_1	l_1	N	%	α_3	β_1	l_1
1	2.3	25	+1 ↖	+1, +2	+2	2.3	27	-3 ←	-2	no	1.9	35	-3 ←	-2	no	1.4	57	-3 ←	-2	no	2.2	47	0 ↓	0, -1	-3
		21	-3 ↖	-3	no		19	0 ↓	0, -1	-3		14	0 ↓	0, -3	no		13	0 ↙	0, -1	-3		33	0, -1 ↙	-1, -3	-3
2	3.3	97	-3 ←	-2	no	3.7	90	-3 ←	-2	no	3.4	97	-3 ←	-2	no	3.0	43	-3 ←	-2	-1	3.8	96	-3 ←	-2	no
		2	-1 ↙	-1,-2	-3		5	-3 ←	-2	no		1	-3 ←	-2	no		24	-3 ←	-2	-1		3	0 ↓	0, -1	-3
3	3.9	60	-3 ←	-2	-1	3.4	65	-3 ←	-2	-1	2.8	47	-3 ←	-2	-1	2.0	36	-1 ↘	-1, -2	-2	3.9	82	-3 ←	-2	-1
		31	-1 ↙	-1,-2	-2		19	-1 ↘	-1, -2	-2		40	-1 ↘	-1, -2	-2		22	-2 ↖	-2	-1		7	-1, -2 ↓	-1, -2	-2
4	2.7	83	0 ↖	+3	+3	2.3	73	0 ↖	+3	+3	1.8	67	0 ↑	+3	+3	1.6	30	+3 ↙	+3	+1	2.6	70	0 ↖	+3	+3
		4	-3 ↖	-2	-1		6	-3 ↖	-1, -2	-1		13	-3 ↙	0, -3	0		29	+3 ↙	+3	+1		11	0 ↑	+3	+3
5	4.0	59	-1, -2 ↗	-1, -2	-3	3.1	60	-1,-2 ↗	-1,-2	-3	2.7	50	-1,-2 ↗	-1,-2	-3	2.2	59	-2 ←	-2	no	2.3	36	-1,-2 ↗	-1, -2	-3
		27	-2 ↖	-2	no		23	-2 ←	-2	no		30	-2 ←	-2	no		25	+1, +2 ↓	+1,+2	0		20	-1,-2 ↗	-1, -2	-3
6	1.9	63	-2 ←	-2	no	1.7	71	-2 ←	-2	no	1.5	72	-2 ←	-2	no	1.1	33	-1,-2 ↗	-1, -2	-3	2.0	35	-2 ←	-2	no
		29	-1,-2 ↗	-1,-2	-3		20	-1,-2 ↗	-1, -2	-3		16	-1,-2 ↗	-1, -2	-3		23	+2, +3 ↙	0, +3	0, +3		18	-3 ↖	-1, -2	-1
7	1.5	49	-2 ←	-2	no	1.7	55	-2 ←	-2	no	1.4	44	-2 ←	-2	no	1.1	45	-1 ↘	-1, -2	-2	1.9	46	-3 ↖	-2	-1
		22	-3 ↖	-2	-1		25	-3 ↖	-2	-1		36	-3 ↖	-2	-1		34	-3 ↖	-2	-1		39	-3 ↖	-2	-1
8	4.0	75	-1 ↘	-1,-2	-2	3.9	59	-2 ↖	-2	no	3.7	50	-2 ↖	-2	no	4.0	26	+3 ↙	0, +3	+1	3.2	47	-2 ↖	-2	no
		8	-1 ↘	-1,-2	-2		24	-2 ↖	-2	no		21	-2 ↖	-2	no		21	+3 ↙	0, +3	+1		11	-1 ↘	-1, -2	-2

References:

1. Zhou, B.-R., J. Jiang, H. Feng, R. Ghirlando, T.S. Xiao, and Y. Bai. 2015. Structural Mechanisms of Nucleosome Recognition by Linker Histones. Mol. Cell. 59: 628–638.

2. Bednar, J., I. Garcia-Saez, R. Boopathi, A.R. Cutter, G. Papai, A. Reymer, S.H. Syed, I.N. Lone, O. Tonchev, C. Crucifix, H. Menoni, C. Papin, D.A. Skoufias, H. Kurumizaka, R. Lavery, A. Hamiche, J.J. Hayes, P. Schultz, D. Angelov, C. Petosa, and S. Dimitrov. 2017. Structure and Dynamics of a 197 bp Nucleosome in Complex with Linker Histone H1. Mol. Cell. 66: 384–397.e8.

3. Öztürk, M.A., G.V. Pachov, R.C. Wade, and V. Cojocaru. 2016. Conformational selection and dynamic adaptation upon linker histone binding to the nucleosome. Nucleic Acids Res. 44: 6599–6613.
